# A Proposed Participatory Framework for Explainable AI in mHealth: Mixed Methods Study Integrating User and Stakeholder Requirements

**DOI:** 10.2196/87158

**Published:** 2026-05-04

**Authors:** Farzana Islam, Ashraful Islam, M Ashraful Amin, Moinul Zaber

**Affiliations:** 1 Center for Computational & Data Sciences, Independent University, Bangladesh, Plot 16, Block B, Aftab Uddin Ahmed Road, Bashundhara R/A, Dhaka, Dhaka Division, 1245, Bangladesh, 880 9612-939393

**Keywords:** artificial intelligence, explainable AI, mHealth, digital health, human-centered design, trust in AI, Global South, responsible AI, clinical decision support, AI transparency, participatory design

## Abstract

**Background:**

Artificial intelligence (AI) integration in mobile health (mHealth) apps offers health care access opportunities in low-resource settings, yet opaque AI recommendations undermine trust and adoption. Existing explainable AI (XAI) frameworks, designed in Western contexts, fail to address the linguistic, cultural, and infrastructural realities of South Asian populations, creating barriers where users cannot understand AI recommendations, clinicians cannot validate outputs, and developers lack implementation guidance. Thus, understanding explainability requirements among educated, digitally literate populations provides foundational insights for future development of inclusive mHealth technologies.

**Objective:**

This study aims to (1) investigate stakeholder perceptions of trust and explainability in AI-driven mHealth in Bangladesh; (2) identify demographic predictors of trust; and (3) develop and propose a context-adapted framework benefiting developers, policymakers, clinicians, and end users in resource-constrained settings.

**Methods:**

This study used a sequential mixed methods design that combined a quantitative survey (n=137) with a qualitative phase involving 20 stakeholders. This qualitative cohort consisted of developers (n=4), XAI experts (n=6), and clinicians (n=10) who participated through either focus groups or individual interviews. We used statistical analysis to examine demographic predictors and applied thematic analysis to identify explainability needs specific to each stakeholder group.

**Results:**

Education level showed a significant effect on trust (*F*_3, 133_=2.81, *P*=.042). Completed undergraduate students reported lower trust (mean 3.14, SD 1.10) compared with current undergraduates (mean 3.66, SD 0.93), suggesting that undergraduate completion develops critical evaluation skills that may decrease uncritical acceptance of AI systems. Despite recognizing AI’s utility for preliminary guidance, users emphasized the necessity of human validation and expressed concerns about understanding AI’s decision-making logic. Interviews with different stakeholder groups revealed critical gaps. Developers acknowledged minimal explainability implementation in current mHealth apps, while medical professionals unanimously prioritized clinical judgment over automated outputs and advocated for physician-mediated AI systems. Synthesizing findings across all stakeholder groups revealed five core requirements: (1) Human–AI collaboration and clinical validation, (2) Transparent logic paths, (3) Contextual personalization, (4) Cultural and linguistic relevance, and (5) Trust calibration and ethical safeguards.

**Conclusions:**

The framework bridges stakeholder misalignments and offers actionable guidance for design, deployment, and policy alignment in resource-constrained environments. By situating explainability within the sociocultural realities of South Asia, this research advances XAI beyond algorithmic transparency toward equity, inclusion, and user empowerment in digital health.

## Introduction

### Background

Artificial intelligence (AI) is the ability of a computer system to learn and make decisions based on what it has learned from a given set of data, and it is transforming almost every aspect of health care [1]. It is now routinely applied to disease prediction, clinical decision support, and population-scale analytics, often outperforming traditional statistical methods. In parallel, mobile health–related apps (mHealth apps) have risen dramatically, offering symptom checkers, medication reminders, and personalized lifestyle advice [2]. The World Health Organization (WHO) reports that digital health adoption accelerated by over 70% following the COVID-19 pandemic, with AI-enabled mobile services showing the fastest growth [3]. The global mHealth market now exceeds 387 million active users, with forecasts surpassing 450 million by 2026, and more than 36,000 health apps available in the Google Play Store [4]. However, as AI-driven systems proliferate, their trustworthiness, explainability, and equity are increasingly questioned [5,6].

AI systems are often described as “black boxes” that generate recommendations without revealing their underlying logic [7]. Explainable AI (XAI) seeks to mitigate this opacity by providing transparent reasoning, feature importance, and confidence metrics [8,9]. Research shows that well-designed explanations can enhance clinicians’ cognitive and affective trust, whereas poor or overly complex explanations can undermine confidence [10]. In mHealth contexts, explainability challenges are amplified by continuous personal data collection, real-time decision-making, and the need to communicate complex health information through small screens to users with differing literacy levels. Unlike clinical AI systems built for professionals, mHealth apps must balance technical accuracy with lay comprehension while delivering health-critical recommendations. The lack of meaningful explanations in such apps has been linked to reduced engagement, poor adherence, and premature abandonment [11,12]. Consequently, explainability is not merely a desirable feature but a prerequisite for safe, effective, and trustworthy AI adoption in mHealth care [6,13].

The global XAI research landscape reveals a striking geographic imbalance. While the field has matured rapidly in high-income countries [14-16], only a few published studies have examined XAI in Global South contexts [17]. This bias has produced frameworks ill-suited for low-resource environments characterized by limited infrastructure, small datasets, and low AI literacy [18,19]. The resulting “AI divide” reflects disparities in investment, institutional readiness, and the cultural relevance of AI technologies [20-24]. Fear and uncertainty further impede AI literacy; global surveys reveal nearly equal proportions of adults expressing nervousness (52%) and excitement (54%) about AI [25]. Without deliberate inclusion, the AI revolution risks widening existing digital and social inequalities rather than bridging them.

Within this global context, Bangladesh exemplifies both progress and persistent challenges. With over 188 million mobile connections and a penetration rate of 108%, the country has achieved remarkable digital connectivity [26]. Yet digital literacy, affordability, and sociocultural barriers continue to hinder meaningful participation in AI-driven health technologies [27,28]. The health care system faces severe workforce shortages, with around 5 physicians and 2 nurses per 10,000 population, with 75% of clinicians serving urban areas where only 38% of people live [29-31]. As a result, mHealth apps often act as the first point of contact for health care advice, especially in rural regions. However, if users cannot understand or trust AI recommendations, the potential of such systems to expand health care access remains unrealized.

The explainability requirements differ between the stakeholder groups in AI mHealth. End users prioritize concise and actionable explanations that foster confidence without technical complexity [32,33]. Developers and data scientists seek diagnostic insights into model behavior and performance to ensure reliability [34,35]. Clinicians demand clinically valid evidence pathways aligned with medical standards [6,27], while policymakers require auditability and ethical accountability [36]. Addressing these divergent needs necessitates a participatory, multistakeholder approach that integrates technical and human perspectives. Human-centered XAI emphasizes designing explanations that align with users’ mental models, cognitive capacities, and cultural contexts rather than exposing raw model mechanics [37]. Personalized, context-aware explanations have proven more effective, particularly among populations with limited digital literacy [38]. In low-resource settings, where mHealth may substitute for clinical care, poorly designed or culturally mismatched explanations can lead to dangerous misinterpretations. Effective explanations must therefore not only inform but also empower users to make safe, contextually appropriate health decisions while maintaining trust and acknowledging uncertainty.

Bangladesh provides a compelling research context for exploring these issues. It exhibits features representative of South Asia, as well as the Global South, an emerging digital health industry, socioeconomic diversity, and varied literacy levels [39,40]. The nation’s demographic and infrastructural patterns mirror those of countries such as India, Nigeria, and Indonesia [41-43], making insights transferable to similar settings. These parallels position Bangladesh as a microcosm for studying how AI explainability can be designed to accommodate constrained resources, cultural diversity, and evolving digital ecosystems.

Although mHealth apps promise to democratize health care, opaque AI components risk undermining their effectiveness, especially where literacy, infrastructure, and trust barriers persist. Most XAI research has been conducted in Western contexts [14,15,44,45], leaving a critical gap in understanding explainability for low-resource users [19,38,46]. Addressing this gap requires empirical evidence on how different stakeholders perceive and require explainability in mHealth apps.

Accordingly, this study aims to investigate stakeholder perceptions of trust and explainability in AI-driven mHealth apps in Bangladesh and to synthesize findings into a human-centered design framework for the South Asian community. While our broader goal is to inform XAI design for diverse populations, including underserved communities, the current absence of Bangla-language AI-based health apps with XAI features necessitated studying English-proficient, educated users as an essential first step. These findings represent the perspectives of digital natives and early adopters in Bangladesh, providing foundational insights that can inform the future development of vernacular mHealth apps for broader population segments, including low-literacy monolingual users.

### Significance and Motivation

This study addresses a critical gap at the intersection of AI, global health equity, and human-centered design. While XAI research has matured in high-income countries, South Asian populations remain underrepresented in both the design and evaluation of XAI systems [17,47]. This geographic imbalance has profound implications for health equity: AI systems trained on Western data and designed for Western users systematically fail to meet the needs of populations who could benefit most from accessible digital health technologies [44,48].

Bangladesh exemplifies the challenges facing AI-driven health care in low-resource settings. The country has widespread mobile connectivity (108% penetration [26]) yet faces severe health care workforce shortages (5 physicians per 10,000 population compared with 36 in high-income countries [29,42]) alongside significant linguistic and cultural diversity.

Three interconnected concerns motivate this work. First, the transparency paradox: while technical XAI methods such as LIME (local interpretable model-agnostic explanations), SHAP (Shapley additive explanations), and attention mechanisms proliferate in research literature [49-51], these tools remain inaccessible to end users and fail to address fundamental questions of trust, cultural appropriateness, and actionability in health care contexts [17,32]. Second, the stakeholder gap: existing frameworks rarely incorporate perspectives from developers facing resource constraints, clinicians navigating local treatment protocols, or users with limited digital literacy [45,47,51]. This results in solutions that are technically sound but practically unusable. Third, the equity imperative: as AI systems increasingly mediate access to health information and services, the absence of explainability adapted to diverse populations threatens to widen rather than narrow global health disparities [18,44,50].

This research is significant for 4 interconnected reasons. Methodologically, it provides rare empirical evidence on XAI requirements from a South Asian context, addressing what scholars have termed “algorithmic colonialism,” or the imposition of Western AI paradigms on non-Western populations [44]. Theoretically, it demonstrates that explainability is not purely a technical or cognitive challenge but fundamentally a sociocultural one, shaped by language, institutional trust, health care norms, and power dynamics [8,50]. Practically, it offers actionable guidance for developers, policymakers, and health institutions navigating the complex terrain of responsible AI deployment in resource-constrained environments. Ethically, it foregrounds the voices of users, clinicians, and developers who are typically excluded from AI design conversations, embodying principles of participatory and inclusive technology development [47,51].

By investigating explainability requirements across multiple stakeholder groups, including users with diverse educational backgrounds, developers with limited resources, clinicians prioritizing patient safety, and XAI experts balancing technical feasibility with human needs, this study generates insights that bridge research, practice, and policy. The resulting framework operationalizes explainability not as an algorithmic property but as an ecosystem of transparency, trust, cultural resonance, and accountability.

### Prior Work

#### Explainable Artificial Intelligence

AI is increasingly integrated into health care, enabling data-driven diagnosis, prediction, and personalized care. Yet, as systems grow more complex, the opacity of their decision-making limits user understanding and trust. XAI addresses this challenge by making algorithmic reasoning interpretable to humans. XAI refers to a collection of procedures and techniques that enable people to understand and have trust in the output and results produced by AI’s machine learning (ML) algorithms. Doshi-Velez and Kim [11] conceptualize XAI as aligning algorithmic logic with human cognition to foster trust and accountability. Similarly, Gunning et al [52] emphasize “glass box” systems that reveal objectives, justifications, and reasoning in ways lay users can comprehend.

Common XAI techniques include post hoc explanation models such as LIME and SHAP, which approximate model behavior for interpretation [49,50], saliency and attention mechanisms that visualize model focus [51], and rule-based surrogate models that simplify complex systems. These methods, however, are primarily designed for technical audiences such as developers and data scientists. Recent scholarship argues for human-centered XAI, which prioritizes usability, accessibility, and contextual appropriateness over technical transparency (Liao et al [53]). Despite this shift, research continues to lack a systematic evaluation of XAI effectiveness for nontechnical users or populations in non-Western, resource-constrained contexts.

#### AI and mHealth: Potential and Pitfalls

AI-powered mHealth apps use ML to support real-time health monitoring, self-diagnosis, and preventive care. These tools expand access to health care, especially in regions where formal medical services are limited. Studies by Deniz-Garcia et al [2] and Singh et al [54] highlight the growing reliance on AI-backed symptom checkers such as Ada, WebMD, and Symptomate, which use natural-language processing and probabilistic reasoning to suggest possible conditions.

Existing reviews have highlighted persistent challenges in ensuring the usability, transparency, and effectiveness of AI-enabled mHealth apps, and researchers warn that premature or blind trust in AI systems may pose ethical and safety risks. Gerdes [34] and Rudin [13] caution that opaque AI in high-stakes health care can yield inappropriate advice and harm patient safety. Beede et al [45] further demonstrate that technically sound AI systems may still fail in real-world deployment due to workflow misalignment, low explainability, and user distrust. Regulatory frameworks for AI in health care remain nascent, leaving uncertainty around accountability and validation. Thus, mHealth apps must balance innovation with transparency and cultural relevance to ensure user safety and equitable access.

#### Trust, Transparency, and Human-Centered XAI

Trust is central to AI adoption in health care. Shin [9] finds that explainability enhances user trust by revealing causes and reasoning behind outputs. Trust depends not only on accuracy but also on how results are communicated. Kizilcec [55] shows that users engage more confidently with systems that disclose their reasoning, particularly when users lack domain expertise.

Ehsan et al [8] advocate “social transparency,” emphasizing narrative and contextual explanations over technical details to humanize AI interactions. This is especially relevant for health care, where patients need empathetic and understandable rationales rather than statistical evidence. Visser et al [12] demonstrate that explanation preferences differ among stakeholders: clinicians seek evidence-based validation, while patients value intuitive, emotionally reassuring feedback. Consequently, one-size-fits-all explanations are ineffective; adaptive and customizable approaches are required.

Participatory design has emerged as a promising approach to bridge these gaps. Liao et al [53] and Larasati et al [32] argue that effective XAI must be co-designed with patients, clinicians, and developers. Engaging stakeholders in the design process ensures that AI explanations meet real-world cognitive, professional, and ethical expectations, thus strengthening user trust and accountability.

#### XAI in South Asia and Global South: Challenges and Contextual Needs

Despite progress in XAI research, most studies are grounded in Global North contexts. Scholars such as Okolo [47] and Sambasivan et al [48] describe this imbalance as a form of “algorithmic colonialism,” where models and design assumptions from high-income countries are exported to environments with distinct cultural and infrastructural realities. AI trained on Western datasets may fail to generalize, such as dermatology models that misdiagnose darker skin tones or provide explanations that are linguistically or culturally inappropriate.

Islam et al [38] show that users in South Asia, including Bangladesh, often interact with AI technologies without fully understanding them due to limited digital literacy. This can lead to both over-reliance and distrust, depending on prior experience. Ismail and Kumar [56] likewise observed among Indian community health workers that sociocultural context, language accessibility, and human mediation are essential for AI acceptance.

Okolo et al [47] emphasize the absence of localized explainability strategies that account for language diversity, cultural norms, and expectations around medical authority. To improve credibility and adoption, XAI systems in South Asia should incorporate:

- Endorsement by local health institutions, linking AI recommendations to trusted authorities.
- Integration of national clinical guidelines, ensuring contextual medical validity.
- Local language and visual support, accommodating low literacy.
- Consideration of drug availability and affordability, enhancing actionability.

Without such adaptation, AI systems risk being viewed as foreign, untrustworthy, or irrelevant. The lack of culturally grounded XAI approaches threatens to widen rather than close global health disparities.

#### Research Gap

The literature demonstrates substantial technical progress in XAI but limited focus on contextual, user-centered design. In health care, where AI recommendations influence real decisions, explanations must be culturally sensitive, transparent, and trust-building. Most existing work, however, reflects Global North priorities and assumes high digital literacy, overlooking the heterogeneous realities of users in the Global South as well as South Asia. Stakeholder voices such as doctors, developers, and patients are seldom integrated into XAI design, creating a critical research gap. This study addresses these limitations by:

- Investigating how users, developers, and health care professionals in Bangladesh, a country in South Asia, perceive and require explainability in mHealth AI systems.
- Identifying design principles that balance technical feasibility with cultural and cognitive accessibility.
- Proposing a participatory, context-aware framework that operationalizes XAI for equitable health care delivery.

By embedding social, cultural, and institutional dimensions into XAI design, this work advances an inclusive, human-centered approach to AI in global health, ensuring that mHealth technologies empower rather than exclude the populations they aim to serve.

### Research Questions and Objectives

Research objectives are as follows: (1) to characterize perceptions of trust and explainability across users, developers, and health care professionals; (2) to evaluate explanation formats suitable for varying literacy levels; and (3) to construct a participatory design framework for explainable mHealth AI.

Research questions are as follows: (1) How do different stakeholders perceive the trustworthiness and explainability of AI-generated health recommendations? (2) What explanation formats and approaches are most meaningful for users with differing literacy levels? (3) What expectations and concerns do developers, clinicians, and experts have regarding explainability in mHealth AI? (4) How can these needs be synthesized into a framework for explainable mHealth AI in low-resource settings?

By situating XAI within Bangladesh’s mHealth ecosystem, this research contributes empirical evidence to an underrepresented region and offers practical guidance to develop transparent, equitable, and reliable AI systems.

### Study Contributions

This research makes 3 primary contributions, as shown in the following sections.

#### Empirical Contributions

- First comprehensive multistakeholder evidence on XAI requirements from a South Asian context (n=157 across users, developers, clinicians, and experts).
- Quantitative evidence that education level (*P*=.042) predicts trust in AI explainability, challenging “digital native” assumptions.
- Systematic documentation of stakeholder misalignments revealing gaps between user needs, developer constraints, and clinical requirements.

#### Theoretical Contributions

The theoretical contributions are as follows:

- Reconceptualization of explainability as a sociocultural construct requiring cultural resonance, linguistic accessibility, and institutional validation, not merely algorithmic transparency.
- Evidence supporting the “social explainability” theory: users prioritize human validation and institutional endorsement over technical interpretability metrics.

#### Practical Contributions

The practical contributions are as follows:

- Five-pillar participatory framework operationalized for resource-constrained environments (human-AI collaboration, transparent logic, contextual personalization, cultural-linguistic relevance, and ethical safeguards).
- Actionable implementation guidance for developers addressing real constraints (limited resources and absence of localized NLP tools).
- Evidence-based for policymakers establishing AI governance standards and clinical validation protocols in health care contexts.

By centering voices from contexts where 3+ billion people share similar infrastructural realities, this work advances responsible AI toward equity, inclusion, and user empowerment in global digital health.

## Methods

### Overview

This study used a human-centered mixed methods approach to explore how explainability can be effectively designed in AI-driven mHealth apps. The aim was to identify what makes explanations clear, trustworthy, and contextually relevant in the sociocultural environment of Bangladesh. By integrating quantitative and qualitative data, the research captured perspectives from end users, health care professionals, app developers, and XAI researchers. A sequential design was used: survey findings informed subsequent interviews, ensuring both breadth and depth in understanding explainability needs.

### Study Design

The study was conducted in 2 phases, each focusing on a distinct stakeholder group.

- User Survey: Explored perceptions of trust, transparency, and explanation preferences among mHealth users.
- Expert and Developer Interviews: Investigated technical and design considerations related to XAI implementation.
- Key Informant Interviews (KIIs): Engaged medical professionals to assess clinical validity, usability, and trust concerns.

**Figure 1.**
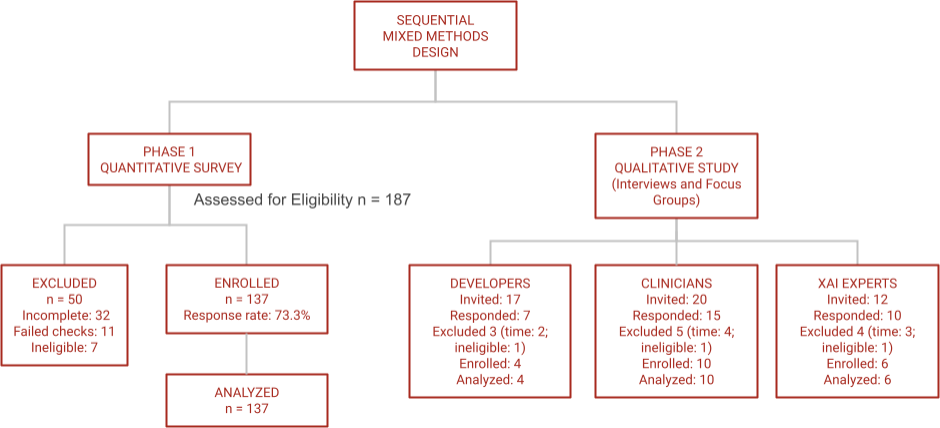
Study design and participant recruitment flow diagram. XAI: explainable AI.

In Figure 1, the detailed recruitment process is shown.

Multimedia Appendix 1 presents an interview guide for stakeholder interviews with developers, clinicians, and XAI experts. Multimedia Appendix 2 presents the survey questionnaires administered to end users.

**Figure 2.**
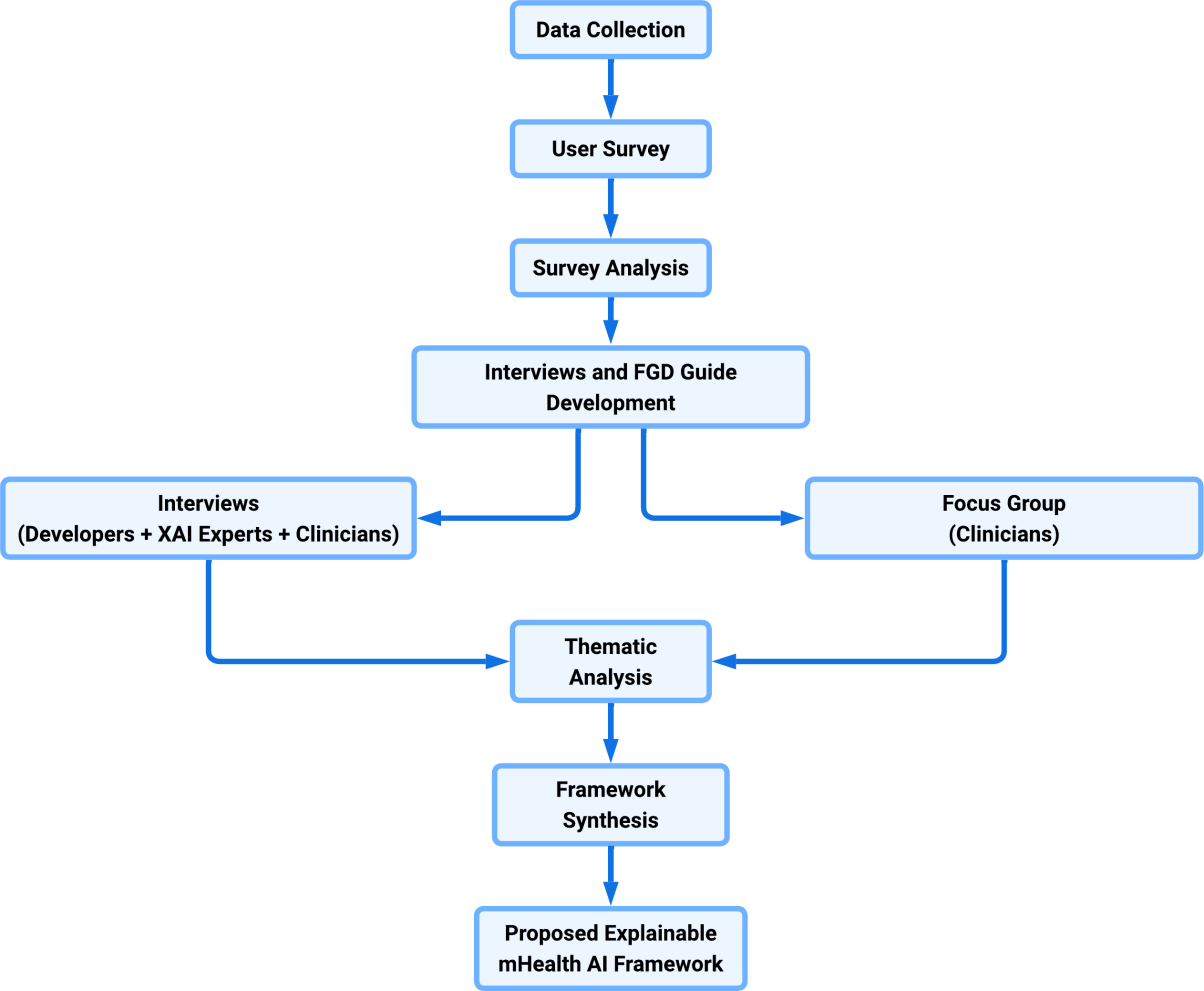
Sequential mixed methods research design used in this study. AI: artificial intelligence; FGD: focus group discussion; mHealth: mobile health; XAI: explainable AI.

Table 1 summarizes participants and methods, and Figure 2 shows the sequential mixed methods design used in this study. Quantitative survey findings informed the development of interview and focus group discussion guides. The qualitative responses were analyzed thematically and integrated with survey insights to develop the proposed explainable mHealth AI framework.

**Table 1.** Summary of study participants and methods.

Stakeholder group	Number of participants	Method	Mode	Duration (mins)	Notes
mHealth^a^ users	137	Survey	Online	15‐20	Used Ada, WebMD, and Symptomate apps
XAI^b^ experts	6	Interview	Online + In-person	30‐40	Global North and South representation
App developers	4	Interview	Online + In-person	30‐40	Local and international mHealth experience
Medical professionals	10 (1+9)	Interview + Focus Group	Online + In-person	45‐60	Practicing in Bangladeshi hospitals

a mHealth: mobile health.

b XAI: explainable artificial intelligence.

### User Survey and Pilot Testing

#### Overview

The user survey examined app usage trends, demographic factors, and perceptions of explainability in AI-driven mHealth apps. We used Google Forms for anonymous online data collection. Participation was voluntary, with informed consent obtained prior to access. The survey instrument was pilot tested with 15 participants matching the target demographic before full deployment. Pilot participants completed the survey after using one of the mHealth apps and provided feedback on question clarity, length, and comprehensibility. Based on pilot results, the survey was revised to simplify technical terminology, reduce redundant items, and clarify instructions. This pilot testing ensured the instrument was appropriate for the target population and capable of capturing meaningful explainability and trust perceptions.

#### Survey Structure

The questionnaire included three parts:

1. Demographics and Background: Collected age, gender, region, education, and AI familiarity to examine how explainability perceptions varied across groups.
2. AI Health App Usage Context: Explored frequency, familiarity, and trust in AI-based symptom checkers.
3. Perceptions of Explainability: Combined 5-point Likert-scale items [57] with open-ended questions [58] such as “Why do you trust or distrust the app’s recommendation?” and “What would make the app’s suggestions more explainable or trustworthy?”

To ensure participants understood the concept of explainability regardless of their familiarity with technical terminology, we implemented a structured approach. After asking whether participants were familiar with “Explainable AI,” all participants received a clear, jargon-free explanation. The explanation described explainability as “the app’s ability to show you WHY it gave a particular recommendation and HOW it reached that conclusion,” using everyday language and culturally relevant health examples. For instance, we explained, “If an app suggests you might have the flu, explainability means it tells you which symptoms led to this suggestion, rather than just giving you the result.” This ensured participants rated actual explainability features (transparency of AI reasoning) rather than general UI satisfaction.

#### App Selection

Three widely used AI health apps, namely Symptomate, Ada Health, and WebMD Symptom Checker [59], were selected based on (1) free availability on both iOS and Android platforms, ensuring broad accessibility; (2) diversity in explanation design: Ada offers Bayesian “white-box” reasoning, Symptomate provides dynamic questioning, and WebMD uses traditional list-based diagnostics [54]; and (3) established user bases with more than 3 million users worldwide. Table 2 gives the comparative analysis of these 3 apps.

**Table 2.** Comparison of selected artificial intelligence (AI) health apps.

Characteristics	Symptomate	Ada Health	WebMD Symptom Checker
User interface	Interactive questionnaire with chatbot functionality	Conversational interface similar to ChatGPT	Traditional list-based approach with text input
Interaction model	Dynamic, personalized questionnaire	Conversational AI with follow-up questions	Symptom selection and body-based navigation
AI architecture	Interactive questionnaire with personalized algorithms	White box Bayesian probabilistic system	Conventional diagnostic algorithm
Explainability level	Moderate - implicit explainability via questioning process	High - white box Bayesian probabilistic reasoning	Low - minimal algorithmic transparency
User base	3+ million users globally	13+ million users worldwide	Extensive (part of WebMD ecosystem)
Geographic reach	Global accessibility	148 countries, 11 languages	Primarily English-speaking markets
Primary features	Symptom analysis, health insights, decision support	Health assessment, telemedicine integration	Symptom checking, condition lookup, doctor finder
Assessment approach	Unlimited symptom input with follow-up prompts explaining logic	Medical dictionary comparison using differential diagnosis	Symptom-to-condition matching with minimal reasoning
Output format	Guided health insights	Personalized assessment reports	Potential conditions list with treatment options
Explainability features	Question flow logic, symptom pathway transparency	Bayesian reasoning display, medical logic explanation	Limited - basic condition information
User manual transparency	Process explanation via user experience	White-box system documentation	Minimal AI process explanation
Regulatory compliance	Standard health app guidelines	FDA AI/ML^a^ framework compliance	Medical information standards
Trust indicators	Process transparency through guided questioning	Clinical validation with explicit medical reasoning	Medical brand credibility over algorithmic transparency
User experience focus	Accessibility and ease of use	Comprehensive health companion	Professional medical information

a ML: machine learning.

#### Participant Recruitment and Sample Characteristics

##### Survey Participants

Survey participants (n=137) were recruited using convenience sampling [60]. All had internet access and smartphones. The majority (93/137, 67.9%) were aged 18‐24 years, and 64.2% (n=88) were male. Educational levels were predominantly undergraduate (121/137, 88.3%). About 94% (n=129) were familiar with AI concepts, while 39.4% (n=54) recognized the term “explainable AI.” Approximately half were first-time mHealth users. Tables3  4 summarize key demographics.

**Table 3.** Age and gender distribution of participants.

Age group (years)	Male	Female	Total, n (%)
18‐24	60	33	93 (67.9)
25‐30	21	11	31 (22.6)
31‐40	6	3	9 (6.6)
Older than 40	2	2	4 (2.9)
Total, n (%)	88 (64.2)	49 (35.8)	137 (100)

**Table 4.** Health app usage patterns among participants.

Usage pattern	Values, n (%)	Implication for study
Regular users	5 (3.6)	Experienced with health apps
Very rare users	66 (48.2)	Limited prior experience
First-time users	66 (48.2)	New to health apps
Total	137 (100.0)	Diverse experience levels

##### Interview and Focus Group Participants

Interview and focus group participants (20 total: 4 developers, 10 clinicians, and 6 XAI experts) were recruited through convenience and snowball sampling. Initial contacts were made through the lead researcher’s professional and academic networks in the Bangladeshi health technology and medical sectors. Subsequent participants were recruited through referrals from initial participants. Participation was voluntary. While no financial compensation was provided, light refreshments (tea and snacks) were offered during interviews and focus groups as a gesture of appreciation.

##### Language and Sampling Considerations

Participants were selected based on the fact that all mHealth apps assessed in this study (Ada, WebMD, and Symptomate) are only available in English due to the lack of Bangla-language mHealth apps with AI and XAI features. Consequently, all participant interactions with these apps occurred in English. This linguistic limitation necessitated recruiting participants with English proficiency, which inherently biased the sample toward educated, digitally literate individuals. The decision to recruit English-proficient participants allowed us to isolate trust and explainability issues stemming from AI opacity and design inadequacies, rather than confounding these factors with basic language comprehension barriers. By ensuring participants could understand the English interface, we aimed to evaluate whether the apps’ explanations were inherently unclear, insufficient, or culturally inappropriate. This approach represents a “best-case scenario” for assessing current international mHealth apps in the Bangladeshi context, as any trust or explainability deficits identified among English-proficient users would likely be substantially amplified among non–English-speaking populations.

### Data Collection

Participants installed and used one app as per their health needs to perform structured symptom-checking tasks before completing the survey. Specifically, participants input their own real-time health data, current symptoms, health concerns, or wellness queries they were genuinely experiencing, rather than hypothetical scenarios. This approach ensured feedback reflected authentic personal experiences. The protocol involved (1) downloading one assigned app (Ada, WebMD, or Symptomate), (2) using the app to check at least one real health symptom or concern, (3) interacting with the AI-driven symptom checker by answering its questions, (4) reviewing the app’s recommendations and explanations, and (5) immediately completing the survey about their experience. This real-time approach captured genuine user perceptions of trust, explainability, and usability that hypothetical scenarios would not provide. Data included quantitative Likert-scale ratings and open-ended qualitative reflections.

### Expert and Developer Interviews

To complement user insights with technical perspectives, semistructured interviews were conducted with 6 XAI experts and 4 app developers. Experts were selected for their work in explainable ML, responsible AI, or human-computer interaction. Developers were practitioners from mHealth companies with experience in deploying AI-based tools in low-resource contexts.

Interviews (30‐60 min) were conducted online or in person using a semistructured protocol. Participants provided informed consent and were anonymized. Topics included limitations of existing XAI tools (eg, LIME, SHAP), trade-offs between accuracy and interpretability, and adaptation of explanations for nontechnical audiences. Developer interviews focused on implementation challenges, interface design, and contextual constraints such as literacy and language barriers.

Interview data were manually coded using an inductive thematic approach. Themes such as technical feasibility, explanation usability, and trust calibration emerged, revealing gaps between research-driven XAI tools and real-world deployment realities.

### The KIIs

KIIs [61] were used to capture clinical perspectives on AI explainability. Ten physicians participated: one in-depth individual interview and a focus group of 9 practitioners from hospitals in Bangladesh. KIIs explored perceptions of AI reliability, trust, patient misuse, and preferred explanation formats.

Sessions were conducted in Bangla (in person or online) and lasted 40‐60 minutes. Doctors expressed cautious optimism toward AI tools but stressed that explanations should align with clinical logic and national treatment protocols. Themes such as clinical trust boundaries, alignment with diagnostic reasoning, and explanation expectations were identified through inductive coding.

### Data Analysis

A mixed methods analytical approach was used, integrating quantitative statistical analysis of survey data with qualitative thematic analysis of interview and focus group transcripts.

### Quantitative Data Analysis

Survey data (n=137) were analyzed using both descriptive and inferential statistics. Descriptive statistics, including means, SDs, medians, and frequencies, were calculated for all Likert-scale items [57] and demographic variables to provide an overview of trust perceptions, explainability ratings, and belief patterns across the sample, summarized in Table 1.

### Inferential Statistical Analysis

To examine differences in trust and explainability perceptions across demographic groups, one-way ANOVA tests were conducted to compare trust ratings (measured on a 5-point Likert scale) across age categories (18‐24, 25‐30, 31‐40, and older than 40 years) and education levels (higher secondary, undergraduate running, undergraduate completed, postgraduate). Given the potential for violations of parametric test assumptions with the sample distribution, Kruskal-Wallis H tests were also performed as nonparametric alternatives to ANOVA for all group comparisons. Both parametric (ANOVA) and nonparametric (Kruskal-Wallis) results are reported to ensure robustness of findings. Effect sizes were calculated for significant ANOVA results using eta-squared (η²), with interpretations following Cohen’s conventions: small (0.01‐0.06), medium (0.06‐0.14), and large (≥0.14) effects. Chi-square tests of independence examined associations between categorical variables, specifically: (1) education level and belief in app decisions (Yes/No/Not sure), (2) XAI familiarity and belief in app decisions, (3) age group and belief in app decisions, and (4) age group and willingness to trust app results over physicians. Statistical significance was set at *α* of .05 for all tests. All quantitative analyses were conducted using Python (version 3.10) with the SciPy statistical library (version 1.9) and Pandas for data manipulation.

### Qualitative Data Analysis

#### Analytical Framework

Open-ended survey responses and interview data were analyzed using Braun and Clarke’s [62] reflexive thematic analysis, following their 6-phase process [63]. This method was selected for its flexibility in capturing diverse stakeholder perspectives while maintaining systematic rigor.

#### Six-Phase Analysis Process

##### Phase 1: Familiarization

All data were read repeatedly to develop deep familiarity. Survey open-ended responses (n=137, in English) and interview notes (in English) were reviewed multiple times. The Bangla focus group transcript was read repeatedly in the original language.

##### Phase 2: Generating Initial Codes

Coding was performed systematically by the lead researcher. English survey responses and interview notes were coded directly. The Bangla focus group transcript was coded in Bangla to preserve cultural-linguistic nuances. A codebook [64] was iteratively developed and refined through peer debriefing with supervisors. Codes captured both explicit meanings (semantic) and underlying assumptions (latent).

##### Phase 3: Generating Initial Themes

Codes were organized into candidate themes by identifying patterns and relationships across the dataset. Visual mapping (tables and clusters) explored connections between codes and potential overarching themes. Preliminary themes were discussed with supervisors who provided critical feedback on theme coherence and boundaries.

##### Phase 4: Reviewing Themes

Candidate themes were reviewed for internal coherence and distinctiveness through regular peer debriefing sessions with supervisors. Coded data extracts were reexamined to ensure adequate support for each theme. Supervisors challenged interpretations and suggested alternative organizations. Themes lacking sufficient data or coherence were revised, merged, or discarded based on this collaborative review.

##### Phase 5: Defining and Naming Themes

Each theme was clearly defined, specifying its scope, boundaries, and relationship to research questions. Theme names and definitions were refined through supervisor feedback. Theme names were crafted to be concise yet evocative.

##### Phase 6: Producing the Report

Final themes were presented with representative quotes. Bangla focus group themes and quotes were translated to English for manuscript presentation (see Translation Pipeline below).

### Translation and Coding Pipeline

The survey was conducted entirely in English, as all evaluated mHealth apps (Ada, WebMD, Symptomate) operate in English. Open-ended survey responses were in English and required no translation.

Individual interviews (n=11: 4 developers, 1 clinician, 6 experts) were conducted in Bangla, with the researcher taking notes in English during interviews to facilitate analysis. One focus group with clinicians (n=9) was conducted in Bangla, audio-recorded, and transcribed verbatim in Bangla by a professional transcriber.

Coding was performed on the Bangla focus group transcript by the lead researcher (native Bangla speaker) to preserve cultural-linguistic authenticity. While initial codes were assigned to Bangla text segments, themes were developed directly in English to facilitate integration with other data sources (English survey responses and interview notes). Representative quotes from the focus group were translated from Bangla to English for manuscript presentation, with back-translation verification (Bangla→English→Bangla comparison) performed to ensure semantic equivalence. We acknowledge the potential loss of cultural-linguistic nuance in this translation process.

### Data Adequacy and Saturation

Formal data saturation assessment (Hennink et al [65]) [66] was not conducted.

Sample adequacy is characterized as:

- User survey responses (n=137): Achieved informational redundancy for primary trust and explainability concerns.
- Developer interviews (n=4): Small sample yields preliminary insights rather than saturated themes.
- Clinician perspectives (n=10 total: 1 interview+9 focus groups): Achieved thematic adequacy for core professional concerns.
- Expert interviews (n=6): Achieved thematic adequacy for technical-ethical considerations.

### Developer Sample Size and Thematic Characterization

While the developer sample was small (n=4), we characterize findings as “themes” rather than “observations” based on several methodological considerations. First, based on the research team’s engagement with Bangladesh’s mHealth ecosystem through professional networks and industry observations, we estimate there are approximately 10 to 15 active mHealth development teams in the country. Our sample of 4 developers, therefore, represents roughly 30% to 40% of this community. However, no comprehensive industry census or registry exists to definitively establish the total number of active mHealth developers in Bangladesh. This estimate should be interpreted as a qualitative assessment of sampling adequacy rather than a statistically validated market share.

Second, we observed strong thematic convergence despite organizational diversity: all 4 developers, working independently across different companies, applications, and technical infrastructures, spontaneously identified similar constraints (resource limitations, lack of Bangla-compatible NLP tools, and market pressures), implementation challenges (explainability deprioritization and technical debt), and design trade-offs.

Third, developer insights were triangulated with other stakeholder findings. User complaints about lack of explanations (n=137), expert observations about implementation gaps (n=6), and clinician concerns about system opacity (n=10) all corroborated developer-reported constraints, strengthening confidence that themes reflected actual industry practices. Fourth, we applied the concept of Malterud et al [67] “information power” rather than traditional saturation criteria, which emphasizes study aim specificity, sample specificity, theoretical application, dialogue quality, and analysis strategy over sample size alone.

Fifth, the 4 developers represented meaningful diversity: different organizational sizes, app types (symptom checkers vs wellness apps), technical approaches (rule-based vs ML), and target populations. However, we acknowledge this sample cannot capture all variations, particularly from larger organizations, international developers, or those with specialized AI/ML expertise. We use “dominant themes” to indicate patterns emerging consistently across our participants within this specific context, not to claim universal prevalence. These findings illuminate current industry constraints in the Bangladeshi mHealth context but require validation with larger, more diverse samples before generalizing beyond this setting.

### Methodological Rigor and Trustworthiness

We used multiple strategies to enhance trustworthiness. Credibility was established through triangulation across surveys, interviews, and focus groups; peer debriefing with supervisors who critically examined interpretations; thick description providing contextual detail; and analysis grounded in extensive illustrative quotes. Dependability was ensured through an audit trail documenting analytical decisions via dated codebook versions, regular progress reports, and meeting notes capturing supervisor feedback. Confirmability was strengthened by grounding findings in participant quotations, actively seeking negative cases (eg, some users preferred simplicity over detailed explanations), and supervisor review of coding and themes. Transferability is supported through a detailed description of the Bangladeshi health care context, participant characteristics, app features, and sociocultural factors, enabling readers to assess applicability to similar settings. Throughout the analysis, regular peer debriefing meetings with supervisors provided critical feedback, questioned assumptions, and challenged analytical decisions, enhancing rigor and reducing individual researcher bias.

### Reflexivity

The lead researcher is a Bangladeshi computer scientist familiar with local health care and technology contexts. This insider status facilitated cultural interpretation and participant rapport but may have led to overlooking aspects obvious to insiders yet notable to outsiders. Technical background aided understanding of developer constraints but required conscious effort to foreground user and clinician perspectives. As a researcher advocating for explainability, I held prior assumptions that transparency improves trust; through reflexive practice and supervisor feedback, I actively sought disconfirming evidence, documenting instances where participants valued simplicity over transparency or expressed concerns that excessive information could overwhelm users.

### Limitation of Single-Coder Approach

While coding was performed by a single researcher, the peer debriefing process with supervisors provided external checks on interpretations. However, the lack of independent intercoder reliability testing remains a limitation. Future research would benefit from dual independent coding with systematic reliability assessment.

### Integration and Triangulation

**Figure 3.**
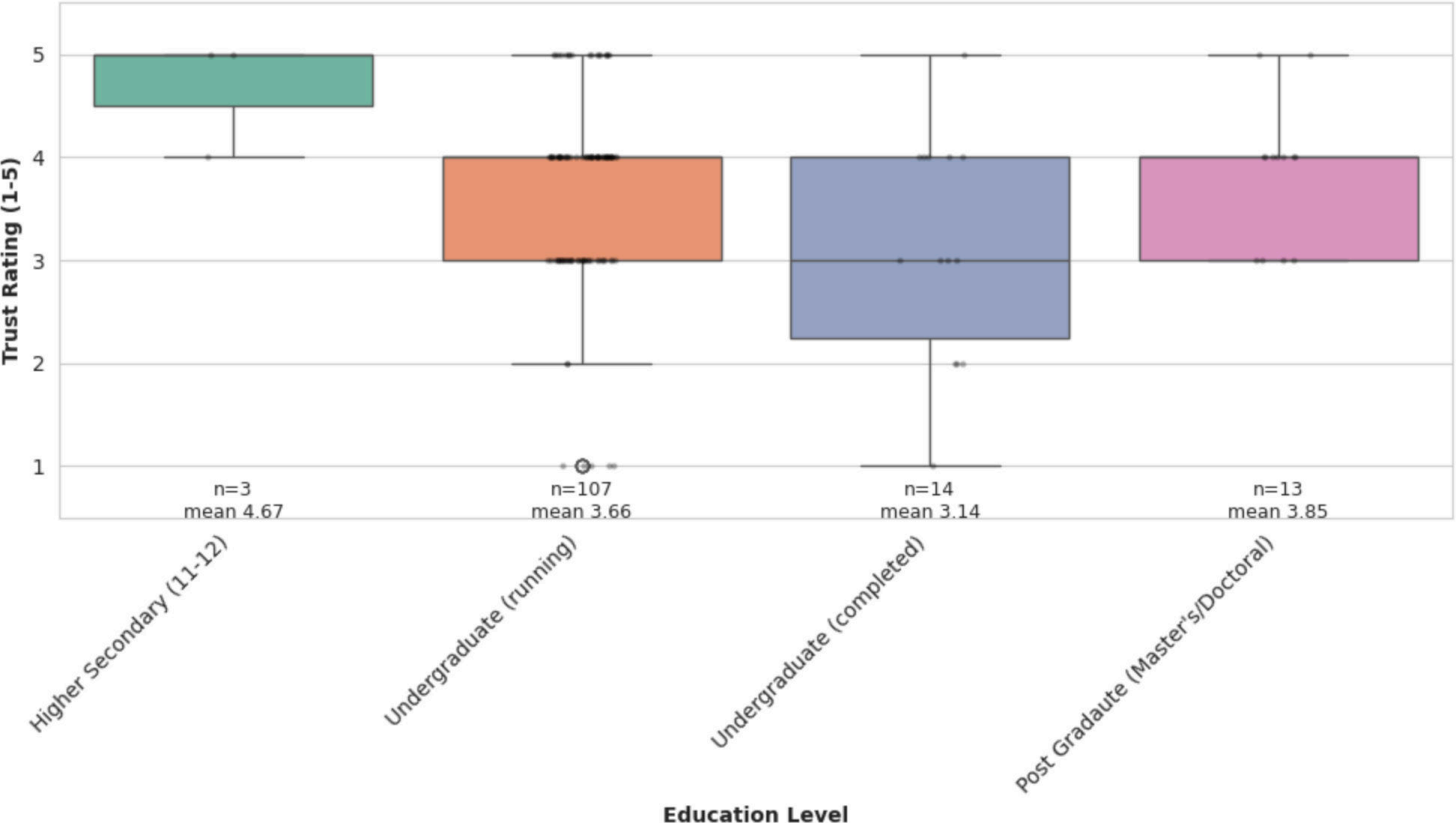
Distribution of trust ratings across education levels.

Triangulation [68] integrated findings across quantitative and qualitative datasets to strengthen validity and develop a comprehensive understanding of explainability needs. Quantitative analysis revealed that education level significantly predicted trust ratings (*P*=.042, Figure 3), while qualitative data provided contextual explanations for why completed undergraduate students expressed lower trust (eg, heightened critical evaluation skills, concerns about AI limitations). User confusion about unclear app logic in qualitative responses paralleled clinician concerns about patient overreliance, while developers highlighted implementation limits of multilingual explanations. Statistical patterns identified in survey data informed the interpretation of qualitative themes, while qualitative narratives provided contextual depth to statistical findings. This cross-stakeholder synthesis informed the development of the proposed XAI framework, ensuring it addressed both measurable trust deficits and nuanced stakeholder concerns.

### Ethical Considerations

This study received full ethical approval from the Institutional Review Board at Independent University, Bangladesh (approval number: 2025-SETS-NSR:03). All research procedures were conducted in accordance with the Declaration of Helsinki and institutional guidelines for human subjects’ research. Care was taken to ensure psychological comfort; sensitive or technical questions were optional.

Beyond procedural ethics, broader concerns were addressed:

- Digital Divide and Equity: To avoid marginalization of low-literacy users, the study emphasized inclusive participation.
- Informed Consent and Trust: Clear language was used to explain study aims and AI-related concepts.
- Algorithmic Bias: Awareness was raised about potential misdiagnosis due to Western-trained datasets.
- Accountability: Discussions highlighted the need for governance mechanisms clarifying responsibility for AI-generated medical advice.

By integrating these principles, the research upheld ethical transparency and participant autonomy throughout all stages of data collection and analysis.

Prior to participation, all participants were provided with a detailed information sheet explaining the study’s purpose, procedures, potential risks and benefits, the voluntary nature of participation, and their right to withdraw at any time without penalty.

For the survey component (Phase 1), participants provided electronic informed consent by checking a box at the beginning of the survey indicating they had read and understood the information sheet and agreed to participate voluntarily.

For interviews and focus groups (Phase 2), participants provided written informed consent prior to data collection. The consent form explicitly informed participants that sessions would be audio-recorded, and participants had the option to decline recording (though none did). Participants were reminded of their right to skip any questions or withdraw from the study at any time.

To protect participant confidentiality, all data were anonymized immediately upon collection. Survey responses were collected anonymously using Google Forms, with no personally identifiable information requested other than the ones mentioned, such as age, gender, etc. Interview and focus group participants were assigned unique pseudonymous identifiers (eg, AD [application developer] 1 for Developer 1, D1 for Clinician 1, P1 for Expert 1) used throughout data analysis and reporting. Audio recordings of interviews and focus groups were stored on password-protected, encrypted devices accessible only to the research team. Transcripts were de-identified before analysis. All data files are stored securely in accordance with Independent University, Bangladesh’s data protection policies and will be retained for (X years, eg, 5 years) as per institutional and regulatory requirements, after which they will be securely destroyed. Screenshot uploads were requested to verify that participants had installed and used the mHealth app. While the survey questionnaire asked users to upload screenshots showing symptom check results, these images were used solely for verification purposes and were not analyzed, coded, or included in any research findings. All uploaded screenshots were securely stored in password-protected cloud storage accessible only to the research team, reviewed solely to confirm app usage, and permanently deleted after verification. No personal health information from screenshots was extracted, recorded, or retained for research purposes.

Participation was voluntary and noncompensated. However, all participants were offered access to study results upon completion. The study posed minimal/no risk to participants. Potential risks included minor discomfort from discussing professional challenges or concerns about AI technologies. Participants were informed they could skip any questions they felt uncomfortable answering and could withdraw at any time. No participant reported adverse events or exercised their right to withdraw during data collection. Contact information for the research team and the Institutional Review Board was provided to all participants for any questions, concerns, or complaints regarding the research.

## Results

### Stakeholder Perspectives on XAI in mHealth

#### User Perspectives on XAI in mHealth Apps

##### Overview

The user study examined how people in Bangladesh perceive and interact with AI-driven mHealth apps, focusing on explainability, trust, and expectations. A total of 137 participants used one of 3 AI-enabled health apps, Ada, WebMD, or Symptomate [59], before completing a survey that included open-ended and Likert-scale questions. The aim was to understand their experiences with app recommendations and their perceptions of the clarity, reliability, and usefulness of explanations.

##### Quantitative Findings: Trust Perceptions Across Demographics

###### Survey Response Overview

A total of 137 valid survey responses were analyzed. Participants were predominantly young adults aged 18‐24 years (93/137, 67.9%), male (88/137, 64.2%), and currently enrolled in undergraduate programs (107/137, 78.1%). Approximately 94% (n=129) reported familiarity with AI concepts, while only 39.4% (n=54) recognized the term “explainable AI.” Overall, participants rated app explainability with a mean score of 3.65 (SD 0.94, median 4.0) on a 5-point Likert scale, indicating moderate trust in app explainability. Trust ratings ranged from 1 to 5, demonstrating substantial variability in user perceptions.

###### Education Level as a Primary Predictor of Trust

One-way ANOVA revealed a statistically significant difference in trust ratings across education levels (*F*_3, 133_=2.81, *P*=.042, η²=0.060), confirmed by Kruskal-Wallis test (H(3)=7.88, *P*=.049). Users with higher secondary education reported the highest trust (mean 4.67, SD 0.58, n=3), followed by postgraduate holders (mean 3.85, SD 0.69, n=13) and current undergraduates (mean 3.66, SD 0.93, n=107). Completed undergraduate students showed the lowest trust (mean 3.14, SD 1.10, n=14). The small effect size (η²=0.060) indicates that education accounts for approximately 6% of the variance in trust ratings, suggesting that other factors also influence perceptions. Figure 3 illustrates these differences visually.

Box plots showing trust ratings (1‐5 Likert scale) across 4 education categories. The box represents the interquartile range (IQR), with the horizontal line indicating the median. Whiskers extend to the minimum and maximum values within 1.5× IQR. Individual data points are overlaid as dots. Sample sizes are as follows: higher secondary (n=3), undergraduate (running; n=107), undergraduate (completed; n=14), and postgraduate (n=13; *F*_3, 133_= 2.81, *P*=.042, η²=0.060 obtained through one-way ANOVA).

###### Age: No Significant Differences

Contrary to initial observations, ANOVA revealed no statistically significant difference in trust ratings across age groups (*F*_3, 133_=0.62, *P*=.60), confirmed by the Kruskal-Wallis test (H(3)=2.72, *P*=.44). Trust remained consistent across ages: 18‐24 (mean 3.63, SD 0.96) years, 25‐30 (mean 3.74, SD 0.89) years, 31‐40 (mean 3.33, SD 0.71) years, and older than 40 (mean 4.00, SD 1.41) years (n=4). While descriptive means vary, differences were not statistically significant.

###### Other Demographic Factors

No significant differences emerged by gender, XAI familiarity, app type, or usage frequency. This pattern indicates that education level, rather than age or other demographics, was the primary predictor of trust differences.

###### Categorical Associations: Belief Patterns

Chi-square tests revealed 2 significant associations. Education level is significantly associated with belief in app decisions (*χ*²_6_=17.08, *P*=.009), with 69% of postgraduate holders believing recommendations compared with 43% of completed undergraduates. XAI familiarity is also significantly associated with belief (*χ*²_4_=11.50, *P*=.02), with 63% of XAI-familiar users expressing belief versus 33% of partially familiar users. No significant associations emerged between age and belief patterns.

###### Language and Cultural Context

It is important to note that these trust ratings reflect user perceptions of English-language apps. Although the participants knew English and could comprehend the app’s content, the language barrier, which is different from AI opacity, may have led to lower trust ratings in a number of ways: (1) reduced cultural resonance of English medical terminology and explanations, (2) increased cognitive load when processing health information in a nonnative language, (3) lack of emotional connection that native-language interactions provide in sensitive health contexts, and (4) contextual inappropriateness of recommendations generated from Western-centric medical knowledge bases. Therefore, observed trust deficits likely reflect a combination of AI explainability gaps and language-cultural barriers.

### Qualitative Findings: User Perspectives

#### Trust and Clarity of Recommendations

Users valued the convenience of mHealth apps for providing preliminary diagnoses but frequently questioned the clarity and trustworthiness of the underlying logic. As one participant explained,

*The app diagnosed me with a condition after I provided my symptoms, but it didn't explain how or why it arrived at this conclusion.*

[P21]

Many participants expressed skepticism about accuracy and a desire for human validation.

*I’m not sure because I’d want a doctor to confirm any treatment.*

[P11]

*I cannot trust what a robot tells me to do.*

[P5]

Participants often viewed AI outputs as uncertain or incomplete, “Sometimes it may not be true” (P4), but believed transparency could mitigate distrust. Users requested explanations that disclosed how recommendations were generated, what data sources were used, and whether clinical guidelines or expert input were applied:

*It would be helpful if the app could provide a brief explanation or reasoning behind its recommendations, including relevant studies or sources.*

[P35]

*The app should provide clear information about the sources of its data and how its AI models work.*

[P53]

These findings reveal a need for explicit, contextually relevant explanations to strengthen user confidence in AI-based health assessments.

#### AI for Early Guidance, Human for Final Judgment

Despite skepticism, most users acknowledged the utility of AI tools as a first point of guidance rather than a replacement for medical expertise.

*The app identified my problem correctly.*

[P6]

*At least it gives me an insight or an idea about what is going on with my health.*

[P39]

Participants described AI as useful for early insight but emphasized the need for human oversight:

*This app might be helpful for decision-making, but you have to go to a doctor finally.*

[P100]

*To make the apps more trustworthy, it could use clear explanations for its decisions and include user feedback to keep improving.*

[P11]

These reflections underscore that explainability enhances perceived utility but does not fully substitute for professional judgment.

#### Preference for Human Judgment

A strong majority preferred human expertise for final decision-making, emphasizing empathy, contextual reasoning, and physical examination:

*Doctors can consider a broader range of factors and provide more personalized insights than an app.*

[P27]

*A doctor can physically examine the situation and give proper treatment, unlike an app that just collects symptom data.*

[P132]

Participants perceived AI as limited in handling rare or complex conditions, as they said, “AI cannot do what humans can do” (P48), reflecting a boundary between computational reasoning and human care.

#### Perceived Benefits and Boundaries of AI Guidance

Participants recognized AI’s analytical strength but remained aware of its limits:

*The app made decisions based on the data, which might be helpful for decision-making.*

[P119]

However, they insisted that transparency and validation were necessary safeguards:

*Transparent explanation of recommendations: The app could provide detailed explanations on how it arrives at its recommendations, including highlighting the key symptoms, medical history, or patterns it considered.*

[P68]

*Explainability features such as providing a rationale for each recommendation can make it easier to trust.*

[P25]

These responses highlight that users accept AI as an assistant rather than an authority and that clear reasoning enhances, but does not guarantee, trust.

#### Differences Among Apps and Familiarity Effects

Although users were randomly assigned one app, differences among Ada, Symptomate, and WebMD shaped perceptions. Chatbot-based systems (Ada, Symptomate) were often viewed as more engaging and transparent than static list-based tools (WebMD). Prior exposure also influenced reactions. First-time users tended to be more forgiving and impressed by novelty, while experienced users were more critical, expecting deeper explanations and medical validation. These variations suggest that interface design and familiarity meaningfully shape how explainability is experienced.

### Improving Explainability Through User-Centered Design

Participants offered constructive recommendations to enhance AI explainability, emphasizing that transparency must be clear, contextual, and human-connected rather than purely technical.

#### Clear and Contextualized Explanations

*Including a clear, concise explanation of the potential risks and benefits associated with each recommendation could make the app’s suggestions feel more reasoned and cautious.*

[P77]

Users desired plain-language rationales that disclose limitations and uncertainties, enabling informed decision-making.

#### Human Oversight and Clinical Endorsement

*A reliable approach would be to offer a paid chat feature that connects users with a live doctor, allowing them to verify whether the app’s results align with a medical professional’s diagnosis.*

[P12]

Participants strongly favored hybrid models combining AI efficiency with clinical validation, aligning with the broader principle of human-in-the-loop explainability.

#### Personalized and Longitudinal Tracking

*The app should include a section to log symptoms and track health records for more personalized guidance.*

[P3]

Customization and data continuity were seen as critical for relevance and user confidence.

#### User Feedback Loops and Adaptive Systems

*Allowing users to provide feedback on whether the recommendations were helpful or accurate could help the AI learn and improve over time.*

[P80]

Participants envisioned bidirectional learning, where user input refines future recommendations and ensures the system stays aligned with local clinical standards.

Overall, users valued AI-driven mHealth apps for their accessibility and informational benefits but were cautious about relying on them without human confirmation. Trust hinged on transparent reasoning, contextual clarity, and human oversight. Explainability was not merely about exposing algorithms; it needed to reflect social credibility, cultural fit, and communicative empathy.

These insights underscore that in South Asia (as well as in the Global South), explainability must move beyond technical interpretability to socially meaningful transparency, where AI recommendations are understandable, verifiable, and situated within trusted human frameworks.

### Expert Perspectives on Explainable AI in mHealth Apps

#### Overview

Interviews with 6 XAI experts (P1-P6) yielded rich insights into the role, limitations, and design priorities of explainability in AI-driven mHealth systems. Participants represented diverse disciplinary and geographic backgrounds spanning AI research, clinical integration, and product design for low-resource settings such as Bangladesh. Thematic analysis revealed several interconnected themes: trust, explanation modality, interpretability and transparency, personalization and contextualization, the role of medical professionals, technical limitations and ethics, and design priorities.

#### Trust as the Cornerstone of Explainability

All experts agreed that user trust fundamentally determines whether AI recommendations are accepted or dismissed. Trust, however, was described as socially situated rather than purely technical. P1 observed that “when people can relate their thinking and the app decision, the user’s trust increases.” P5 added that trust grows when recommendations are “short, reference-based, and clearly attributed to professionals, like- ‘doctor said it, so people trust it.’”

Several experts linked trust to repeated accuracy and social validation. P4 noted that even clinicians “begin to trust tools like GPT after seeing multiple times correct results.” Yet, the contextual fragility of trust was evident in P3’s account: “In Bangladesh, especially low-literate people do not trust doctors… so how can this group trust AI apps?” Trust was therefore portrayed as a fragile construct influenced by professional reputation, algorithmic credibility, prior experience, and broader social attitudes toward health care.

#### Preferred Forms of Explanation

Experts emphasized that effective explainability depends on how information is communicated. Participants favored concise, reference-supported, and context-sensitive explanations. P1 suggested highlighting relevant data: “It should show the relevant data part, like for COVID.” P2 added that explanations should cite evidence: “Just showing the problem is not enough; references should also be given.”

Multimodal presentation was strongly advocated. Visual explanations, such as CAM-based overlays or color-coded graphs, were recommended by P1, P2, and P5 to simplify complex model outputs. P6 proposed “videos or images” for low-literacy users, while P4 recommended “case-study-based explanations” and conversational, narrative formats that mimic physician dialogue. Comparing GPT interactions with brief doctor consultations, P4 noted, “A doctor gives five minutes, but I can talk with GPT for an hour.”

These perspectives indicate a preference for dialogue-driven, story-like explanations that engage users emotionally and cognitively, rather than abstract technical justifications.

#### Interpretability and Transparency

Participants distinguished between interpretability as understanding how the model functions and explainability as understanding why it produced a given output. P1 stressed that most mHealth apps “fail because their internal decision-making is opaque.” They argued that interpretability requires insight into the model’s structure and data sources: “Explainability is based on which data.”

Experts also cautioned that existing tools, such as LIME and SHAP, remain developer-centric. P1 stated bluntly, “They are for developers, not for users.” P3 highlighted “feature-changing issues and bias” as obstacles to consistent interpretation.

Transparency was universally seen as beneficial yet context-dependent. P1 remarked, “People believe what they can understand by nature,” while P5 added, “If the app shows how, it reaches its conclusion, that increases trust.” However, P1 warned that excessive or poorly framed transparency could backfire in misinformation-prone environments: “This is directly related to taboo. For misinformation, social media plays a role.”

Overall, experts advocated layered transparency, offering high-level summaries for general users and deeper interpretive options for clinicians or developers.

#### Personalization and Contextualization

All experts emphasized the importance of tailoring explanations to user literacy, language, and context. Contextual data such as GPS, demographics, and prior history were viewed as crucial for both diagnostic accuracy and relevance. P4 illustrated this need: “In the query, I have to provide the context, like GPS, profile, all in one, to get the correct result.”

P5 proposed a 2-step communication model: first, deliver the analytic result, then supplement it with a context-aware explanation that accounts for individual circumstances. P1 similarly suggested offering “multiple options in a single app” to accommodate diverse user backgrounds.

Inclusivity emerged as a recurring concern. P1 noted a “major opportunity to work with illiterate people,” while P3 cautioned that most current systems are “made for high-rich people,” excluding low-income and low-literacy groups. Experts thus framed personalization not merely as a usability enhancement but as an ethical imperative for equitable AI.

#### Integrating Medical Professionals

Experts strongly endorsed involving clinicians as validators and communicators within AI systems. P1 proposed that “app developers can hire doctors to validate,” and P4 envisioned “a live server connection with a doctor after several conversations.”

Several participants (P2, P5, P6) advocated for embedded live-chat features with health care professionals to enhance trust. As P5 stated, “If a doctor says it, people trust it more; if a developer says it, no one will.” P6 described this feature as promising, though logistically challenging.

Interestingly, P4 observed that XAI could also fill gaps in clinical communication: “In Bangladesh, doctors don’t explain much. If the app can explain properly, it will close this gap and increase trust.” These insights support the notion of human-AI collaboration, where explainable systems complement rather than replace professional judgment.

#### Technical Limitations and Bias

Participants openly discussed the technical fragility of current mHealth AI systems. Bias in training data, feature drift, and limited generalization were cited as major causes of diagnostic error. P3 referred to persistent “feature-changing issues” and “biased results that make explanations difficult.”

Several experts described failure cases, such as misdiagnoses in fracture detection:

*AI made the wrong detection in the fracture case. doctors use cumulative knowledge, but apps use only trained data.*

[P1, P2, P3]

P6 criticized generic outputs like “These are all possible causes; seek medical help,” arguing that such vague responses undermine informed decision-making.

Experts thus underscored the need for robust datasets, domain adaptation, and explainability techniques that acknowledge uncertainty rather than overstate confidence.

#### Ethics, Privacy, and Governance

Concerns over privacy and ethics were universal. Experts stressed that user satisfaction should never compromise confidentiality. As P4 asserted, “Confidential information can’t be shared.” P1 acknowledged regulatory progress, “rules are updating regularly,” while P3 added, “If the company maintains privacy, then it’s okay.”

However, reliance on company-level policies was viewed as insufficient; participants emphasized the need for clear governance, data use, accountability, and regulatory enforcement. The discussions revealed that ethical explainability, making not only decisions but also data practices transparent, is crucial to sustaining public trust.

#### Design Priorities for Inclusive and Actionable Explainability

Experts called for participatory, feedback-driven design that accommodates varied literacy levels and promotes ongoing engagement. P5 urged developers to “deploy the system to minor groups and see their thinking,” highlighting participatory evaluation. P6 pointed to missing feedback loops: “Every hour the app says drink water, but it never asks if I had water later.”

Recommendations converged on several principles:

- Interactive feedback mechanisms so users can confirm, question, or rate recommendations.
- Multioption, inclusive interfaces accommodating text, audio, and visual modalities.
- Localization through language support, cultural cues, and regionally relevant medical references.
- Purpose-specific design, ensuring explainability features match the clinical or advisory scope of the app.

While some experts prioritized data-driven transparency (P1, P2), others favored narrative and conversational forms of explanation (P4, P6). This divergence underscores the need for adaptive explainability, offering multiple modes suited to both lay users and professionals.

### Developer Perspectives on Explainable AI in mHealth Apps

#### Overview

Interviews with 4 AI application developers (AD1-AD4) provided practical insights into the feasibility, value, and challenges of implementing XAI in mHealth apps within Bangladesh. Developers reflected on technical limitations, contextual barriers, and design priorities shaped by local infrastructural and user constraints. Eight dominant themes emerged: developer skepticism and need for validation, gaps in explanation features, designable explanation mechanisms, traceability and transparency, user-centric adaptation, localization barriers, security and data control, and the feasibility of hybrid human-AI systems.

#### Developer Skepticism and Human Validation

Across all interviews, developers expressed conditional trust in AI-generated health recommendations. They considered AI a useful assistive tool but insisted that outputs must be validated by medical professionals. AD1 emphasized that guidance is “reliable and acceptable if doctors verified,” while AD3 admitted, “As developers, we don’t trust AI fully; we verify.”

AD2 noted that “AI hasn’t reached expert doctor level” and should therefore be restricted to “normal situations.” Even AD4’s neutral comment that AI is “good at performing work” lacked endorsement of its medical reliability. Collectively, developers framed trust as contingent upon expert validation, reinforcing the need for human oversight in AI-driven health systems.

#### Gaps in Explanation Features

Developers uniformly acknowledged that explainability remains largely absent from current mHealth apps. AD1 candidly stated, “Explainability-focused work was not done,” while AD2 added, “No explanation-based app I made.” AD3 observed that most apps provide only “minimal explanation,” and AD4 confirmed inconsistency: “Some apps give hints or descriptions; some don’t.”

These remarks reveal that explainability is often deprioritized during development, overshadowed by core functionality and time constraints. As a result, most deployed mHealth systems in Bangladesh lack consistent or meaningful explanation features, leaving users with a limited understanding of how AI-generated advice is produced.

#### Designable Explanation Mechanisms

Despite current gaps, developers proposed actionable strategies to improve XAI integration. Their suggestions emphasized multimodal, interactive, and context-sensitive explanation systems. AD1 recommended incorporating “LLM-based reasoning” to generate natural-language explanations that feel human and conversational. AD2 proposed a tiered design with “a button to expand features” and options for “short or long answers” supported by external resources.

AD3 described an ideal interface that “shows how the decision was made, visuals, text, video, or audio,” while AD4 advocated contextual modality: “During morning walk, I prefer audio; other times maybe visuals.” They also noted that “the image is good, but text should also be included.”

Together, these ideas point to interactive and multimodal explainability, where users can select how and when explanations are presented, balancing cognitive load with accessibility.

#### Traceability and Transparency

Transparency was recognized as essential for both user trust and developer confidence. AD1 simply asserted “transparency” as a trust factor, while AD4 confirmed, “Yes, I want to know,” when asked if visibility into model reasoning mattered. Developers viewed transparency as a shared requirement across user and technical domains; it enables debugging, verification, and accountability.

This emphasis on traceability suggests that explainability must function not only as a user interface feature but also as an engineering principle, helping developers monitor model behavior and assure quality in sensitive contexts such as health advice.

#### Customizable and User-Centric Design

Developers favored adaptable systems that personalize explanations based on user context and literacy. AD3 summarized this vision: “Users can choose what type of explainability they want.” Similarly, AD2 emphasized tailoring output “based on user level” and noted that explanation mechanisms should “improve over time” through user interaction.

AD4 added that explanations could be filtered by situational context to avoid overwhelming users. These perspectives converge on a model of adaptive explainability, where AI dynamically adjusts the complexity and modality of its explanations to fit the user’s background, language, and situational needs.

#### Localization and Usability Barriers

All developers highlighted the importance of localization for mHealth adoption. Language diversity, digital literacy, and socioeconomic factors were identified as key constraints. AD1 recommended “multiple languages and tutorials needed” for onboarding, and AD2 argued that systems must reflect “how people understand local healthcare.”

Financial accessibility also emerged as a concern. AD4 proposed a donation-based model: “Affordability should be addressed. Donations from those who can afford it will support those who can’t.”

These reflections underscore that effective explainability cannot be detached from cultural and economic realities; it must be embedded in an inclusive design that aligns with local capacities and norms.

#### Security, Device-Level Control, and Data Integrity

Developers expressed awareness of privacy and data security challenges, particularly regarding remote processing. AD1 advocated “on-device models” with “encrypted data,” while cautioning that “before explainability, we need to ensure awareness among users.”

AD3 warned that “API can be intercepted,” underscoring risks in cloud-dependent architectures. By contrast, AD4 claimed “no privacy concerns,” reflecting either overconfidence or limited exposure to data governance issues.

Collectively, these insights highlight the dual nature of security in mHealth XAI: it is both technical (encryption, on-device processing) and social, requiring user awareness and consent.

#### Feasibility of Human–AI Hybrid Systems

Developers diverged sharply on whether integrating medical professionals into AI workflows enhances or undermines system value. AD1 advocated hybrid models in which “doctors are in the loop, governed, and revenue given.” AD3 similarly envisioned “initial diagnosis from doctor, follow-up via app,” conditional on sustainable funding.

Others were skeptical. AD2 asked, “If I need to invite professionals, why use AI at all?” questioning the efficiency of hybrid systems. AD4 raised concerns about authenticity and liability: “We need to verify the actual doctor is behind the system. Developers won’t take responsibility if it’s fake.”

These conflicting views illustrate ongoing tension between automation and professional oversight, with feasibility hinging on governance, authentication, and clear role boundaries.

### Key Informants’ Perspectives on XAI in mHealth Apps

#### Overview

Insights from one in-depth interview and a focus group with 9 practicing physicians (D1-D10) in Bangladesh revealed cautious optimism toward the use of XAI in health care. While doctors recognized the potential of AI for clinical assistance, they emphasized that its use must remain under human supervision. Nine interrelated themes summarized their perspectives on trust, safety, localization, and explanation design.

#### Conditional Trust and Clinical Authority

Doctors expressed conditional trust in AI, consistently prioritizing clinical judgment over automated outputs. As one participant noted,

*Even if AI gives a correct prediction, I will rely on my own judgment.*

[D1]

Participants recounted errors such as misdiagnosed ECGs or inaccurate symptom assessments, which eroded confidence. Several warned that conflicting outputs between AI and physicians could confuse patients and undermine professional credibility:

*If the app gives something different from what I say, the patient may think I’m wrong.*

[D7]

Trust, therefore, hinged on AI complementing, not competing with, human expertise.

#### AI as Assistive, Not Autonomous

All participants viewed AI as a supportive tool rather than a replacement for clinical reasoning. It was seen as useful for tasks such as dosage verification and chronic disease monitoring, but unsafe for unsupervised use by patients. As D4 stated, “It can assist doctors, but for users, it is not feasible. They might get overwhelmed.” This reflects a preference for physician-mediated AI, where apps serve as background aids for efficiency and reminders rather than independent diagnostic systems.

#### Deficits in Explanation and Reasoning

Doctors criticized current mHealth apps for lacking clear or actionable explanations. D3 remarked, “If I don’t already know the topic, how will I judge if AI’s answer is right?” While language models like ChatGPT could reference guidelines when prompted, most mobile apps offered opaque outputs with no interpretive reasoning. Explanation quality was thus linked to user expertise by limiting its accessibility to both nonspecialist clinicians and lay users.

#### Dependence on Input Quality and User Literacy

Participants underscored that AI reliability depends on the precision of user inputs, which most patients cannot provide. As D3 explained, “Chest pain could mean ten things; only a doctor can tell which by asking follow-up questions.” Several doctors advocated for access restrictions or literacy-based user segmentation, arguing that open public use could heighten misinterpretation risks.

#### Cultural and Behavioral Barriers

Low public awareness of AI and entrenched cultural beliefs were cited as major barriers to adoption. “Most people don’t know what AI is; that’s why they don’t use it,” said D1. Others noted that people who distrust allopathic medicine were deemed unlikely to trust AI-driven advice to start medication in this direction, reflecting how social beliefs influence technology acceptance.

#### Localization and Contextual Relevance

Strong consensus emerged that Western-trained AI models fail to reflect Bangladesh’s medical realities. D1 observed, “Lesions in BD patients are different; AI trained on Western data won’t work.” Participants stressed that systems must incorporate local disease profiles, drug availability, treatment costs, and national clinical guidelines to build trust and usability.

#### Governance and Ethical Oversight

Institutional endorsement was viewed as essential but insufficient on its own. “If the government released it, I’d compare its output with my own judgment,” said D1, while D9 added, “Even if endorsed, the app must be for doctors, not for patients.” Physicians called for strict regulation, data accountability, and clear boundaries defining who may use and interpret AI outputs.

#### Human-AI Collaboration and Live Consultation

Doctors generally supported live chat or hybrid systems combining AI with human input for mild or follow-up cases. However, they cautioned that remote advice cannot replace physical examination:

*Only by talking, without examining, can we diagnose.*

[D8]

Compensation and verification mechanisms for participating doctors were seen as essential to sustain such systems.

#### Preferred Explanation Design

Participants favored concise, prioritized outputs that present ranked differential diagnoses linked to guidelines and sources. As D3 urged, “Don’t give 10‐20 diagnoses; show what’s most likely and explain why.” Clinically structured, evidence-linked explanations were considered necessary to support both doctors’ decision-making and patient understanding.

Overall, medical professionals characterized explainability as a clinical, not merely a technical, requirement. They envisioned AI as a supervised decision-support tool grounded in localized data, professional validation, and clear communication of uncertainty. Explainability, from their perspective, is essential for ensuring safety, contextual accuracy, and maintaining public trust in digital health systems.

### Stakeholder Gap Analysis: Misalignments in XAI Expectations for mHealth Apps

#### Overview

This analysis integrates insights from users, developers, XAI experts, and medical professionals to identify where expectations diverge regarding XAI in mHealth apps. Although all stakeholders recognize the importance of explainability for trust and adoption, their perspectives differ sharply in validation standards, explanation preferences, and perceived responsibility. Understanding these misalignments is essential for designing inclusive, context-appropriate XAI systems.

#### Trust and Validation

Trust remains the most consistent yet variably defined factor across stakeholders. Users often express willingness to accept AI recommendations when accompanied by simple reasoning or credible sources (eg, “helpful if the app could provide reasoning, including relevant studies” [P35]). In contrast, medical professionals adopt a far stricter stance: “Even if the AI gives a correct prediction, I will rely on my own judgment” (D1), underscoring the clinical accountability gap between end users and physicians. Developers and experts align more closely, viewing trust as contingent on transparency and iterative validation (AD3; P1). However, developers emphasize practical verification (“we don’t trust AI fully, we verify”) rather than the epistemic or cognitive alignment valued by experts.

#### Explanation Depth and User Segmentation

Stakeholders differ in how complex explanations should be. Users prefer brief, plain-language rationales describing potential risks and benefits (P77), while experts advocate multimodal or narrative explanations (eg, case-based or visual) (P2, P4). Developers propose user-selectable formats, “short or long explanations” (AD2) and adaptive delivery “based on user level.” Medical professionals, however, argue that explainability should be contingent on user capability:

*Apps should restrict who can use them based on education.*

[D6]

These perspectives reveal that technical flexibility alone cannot solve the deeper issue of cognitive diversity across audiences.

#### Technical Implementation and Usability

Experts critique existing explainability tools such as LIME and SHAP as “for developers, not for users” (P1), whereas developers acknowledge that “explainability-focused work was not done” (AD1). This indicates a persistent translation gap between technical affordances and user-centered design. While users value understandability, developers confront resource constraints, making advanced interpretability difficult to operationalize. Hence, current mHealth AI systems often underdeliver on transparency despite available techniques.

#### Human–AI Collaboration and Accountability

All stakeholder groups endorse human involvement but differ in form and feasibility. Users favor AI-guided systems with “paid chat features that connect with live doctors” (P12). Experts also recommend doctor-in-the-loop approaches (P1), while physicians express caution, like “Only by talking, without examining, we can’t diagnose” (D8), stressing physical evaluation as indispensable. Developers, however, question the practicality of hybrid models: “If professionals are required, why use AI at all?” (AD2). Responsibility and liability further complicate collaboration: doctors refuse accountability for unverified AI advice, and developers disclaim legal responsibility (“developers won’t take responsibility if it’s fake” [AD4]). This shared reluctance leaves accountability diffuse, a challenge for regulatory integration.

#### Localization and Cultural Context

Localization emerged as a shared priority, but with different emphases. Users implicitly expect culturally aligned recommendations, while experts advocate context-aware features integrating GPS and profile data (P4). Developers focus on multilingual interfaces and affordability (AD1 and AD2), and medical professionals emphasize clinical relevance:

*Lesions in BD patients are different; AI trained on Western data won’t work.*

[D1]

Misalignment here reflects different interpretations of “localization”: for experts and developers, it is technical adaptation; for clinicians, it is diagnostic and therapeutic validity.

#### Privacy, Governance, and Sustainability

Privacy received uneven attention: experts stressed ethical handling of “confidential information” (P4); developers were split between advocating encrypted, on-device models (AD1) and indifference (AD4); doctors emphasized institutional oversight rather than data protection. Economic sustainability also revealed friction: users expect free services, doctors require compensation for hybrid systems (D10), and developers proposed donation or subsidy models (AD4). This underscores that ethical and financial sustainability are as crucial as algorithmic transparency.

Table 5 provides a consolidated view of stakeholder perspectives across key explainability dimensions. It highlights both overlapping concerns, such as the demand for contextualized explanations, and critical misalignments, particularly around trust, responsibility, and economic feasibility.

**Table 5.** Stakeholder perspectives on key explainable AI (XAI) dimensions in mobile health (mHealth) apps.

Dimension	Key insights	Representative quotes / Examples	Stakeholders
Trust in AI^a^	Users accept simple explanations; doctors require clinical validation; developers verify; experts stress alignment with human reasoning.	“Helpful if the app could provide reasoning... including relevant studies.” [P35] “Even if AI gives a correct prediction, I will rely on my own judgment.” [D1] “As developers, we verify.” [AD3]	Users, Doctors, Developers, Experts
Explainability format	Users prefer clear text; doctors need structured logic; developers rely on UI^b^ features; experts suggest visual and narrative formats.	“Clear, concise explanation of potential risks and benefits.” [P77] “Case study-based explanations...” [P2] “Provide prioritized differential diagnoses.” [D3]	Users, Experts, Doctors, Developers
Personalization and context	Experts suggest context-aware systems using GPS and profile data; developers mention adaptive explanation levels; doctors highlight age/literacy needs.	“Apps should restrict use based on age or education.” [D6] “Explanation delivery should improve over time.” [AD2] “Include GPS, profile to get correct result.” [P4]	Doctors, Developers, Experts
Explainability tools gap	Tools like LIME^c^ and SHAP^d^ are not accessible to end users; developers admit little use of explainability tools.	“They are for developers, not for users.” [P1] “Explainability-focused work was not done.” [AD1]	Experts, Developers
Human-AI collaboration	Users favor doctor-in-the-loop models; experts support it; doctors warn about lack of physical exam; developers see conflict with AI autonomy.	“Offer paid chat feature with live doctor.” [P12] “Only by talking, without examining, we can’t diagnose.” [D8] “If I need professionals, why use AI?” [AD2]	Users, Doctors, Developers, Experts
Responsibility and liability	All stakeholders avoid full accountability for AI errors; doctors want clear validation; developers worry about legal risks.	“Developers won’t take responsibility if it’s fake.” [AD4] “I won’t be held liable without proper validation.” [D3]	Doctors, Developers
Localization and cultural fit	Doctors cite mismatch between AI training and local clinical conditions; developers focus on language/UI; experts urge regional adaptation.	“Lesions in BD patients are different, AI trained on Western data won’t work.” [D1] “Understand how people interpret local healthcare.” [AD1]	Doctors, Developers, Experts
Privacy concerns	Users rarely mention it; developers split on its importance; doctors prioritize clinical responsibility; experts warn about ethics.	“Confidential information can’t be shared.” [P4] “On-device model, encrypted data.” [AD1]	Users, Experts, Developers, Doctors
Cost and sustainability	Users expect free access; developers suggest donation models; doctors require fair compensation; experts rarely discuss economics.	“Doctors need remuneration if we’re asked to be involved.” [D10] “Donation model for those who can’t pay.” [AD4]	Users, Developers, Doctor

a AI: artificial intelligence.

b UI: user interface.

c LIME: local interpretable model-agnostic explanations.

d SHAP: Shapley additive explanations.

Overall, while all stakeholders converge on the value of explainability, they diverge on what “good explanation” means in practice. These tensions highlight that effective XAI design in health care must be co-created with all actors, balancing technical transparency with human judgment, cultural nuance, and systemic accountability. This analysis directly informs the framework proposed in the following section, which aims to reconcile these gaps through participatory, context-sensitive design principles.

### Proposed Framework for Explainable AI in mHealth Apps

#### Overview

**Figure 4.**
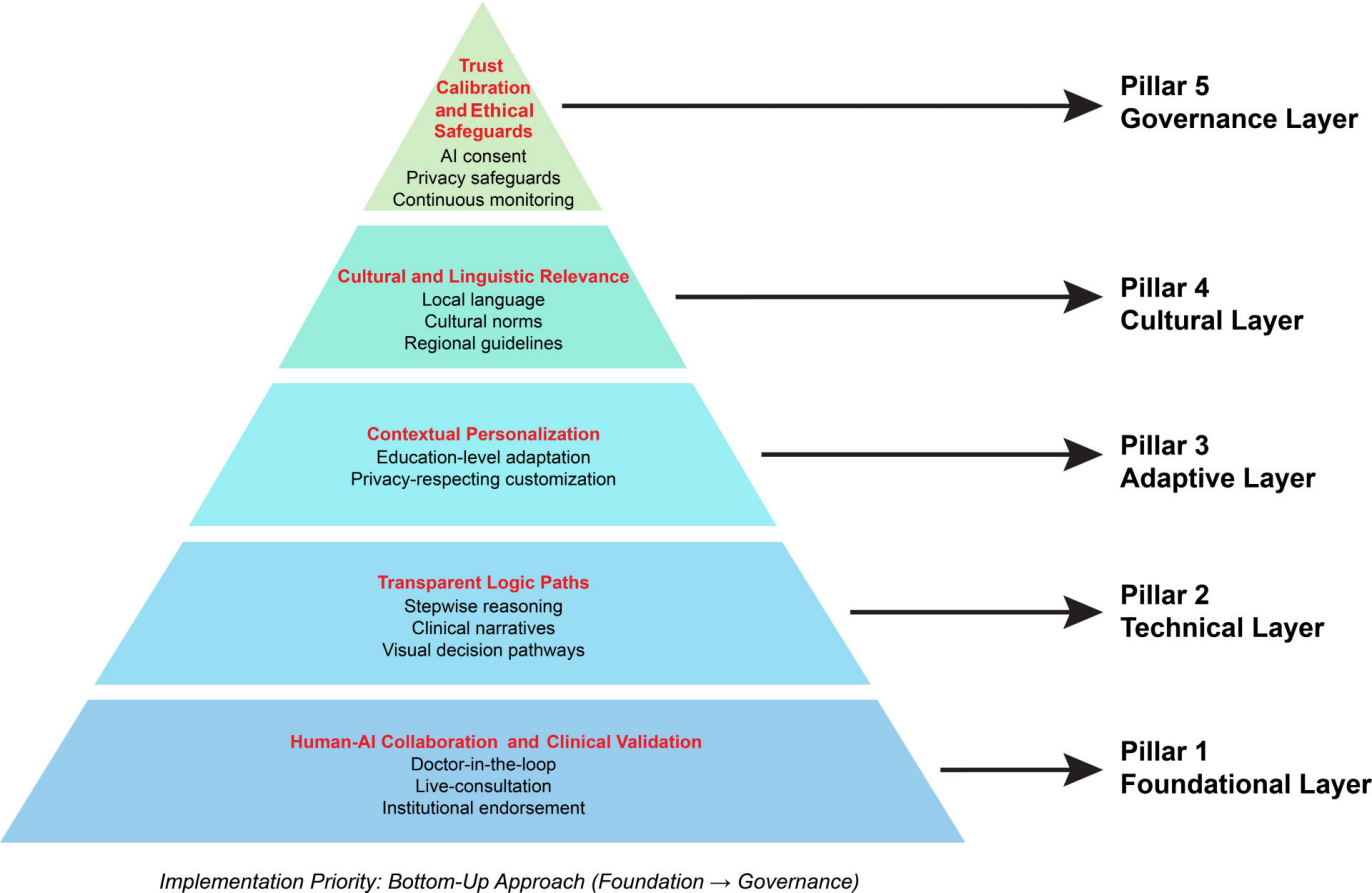
Hierarchical proposed framework for explainable mHealth apps. AI: artificial intelligence; mHealth: mobile health; XAI: explainable AI.

Building upon insights from various stakeholders, we developed a conceptual 5-pillar framework for explainability in AI-driven mHealth apps (Figure 4). The framework positions explainability as a dynamic process emerging from interaction among human understanding, algorithmic transparency, and institutional accountability.

#### Pillar 1: Human-AI Collaboration and Clinical Validation

All stakeholder groups prioritized human oversight over autonomous AI decision-making. Medical professionals emphasized that clinical reasoning must remain the final arbiter of health recommendations. Users preferred apps offering access to professional consultation for validation. Developers and experts endorsed “doctor-in-the-loop” models integrating live chat, institutional endorsement, or verification mechanisms to anchor trust and mitigate overreliance risks.

#### Pillar 2: Transparent Logic Paths

Participants sought clarity about how AI systems reached conclusions, valuing stepwise cause-and-effect reasoning over abstract confidence scores or probability distributions. Users wanted narratives mirroring clinical thought processes (eg, “you reported these symptoms; therefore, these conditions are likely”) rather than technical explanations. This principle requires intuitive reasoning traces, visual summaries, or simplified decision pathways, making the model’s internal logic observable.

#### Pillar 3: Contextual Personalization

Users expected AI systems to reflect personal health profiles, local treatment access, and daily routines. Developers emphasized that personalization enhances perceived relevance and trust when privacy is safeguarded. The framework envisions adaptive explanation systems adjusting depth, tone, and modality (text, visual, and audio) based on user literacy, familiarity, and prior interaction as identified through stakeholder consultation. Stakeholders emphasized that explainability requirements vary substantially across users, with personalization extending to clinical adaptation considering region-specific disease prevalence, local drug availability, and affordability.

#### Pillar 4: Cultural and Linguistic Relevance

Users and clinicians highlighted how Western-centric terminology, English-only interfaces, and unfamiliar medical constructs can alienate users. Trust depends on linguistic and cultural resonance. This principle requires explanations in native languages supported by voice narration, culturally familiar metaphors, examples from local health practices, and integration of national clinical guidelines and approved medication lists.

#### Pillar 5: Trust Calibration and Ethical Safeguards

Users expressed privacy concerns, while experts emphasized communicating AI limitations clearly. This pillar advocates for explicit confidence indicators (“high certainty” and “low data confidence”), clear data use disclosure, visible ethical endorsements from professional or governmental bodies, feedback mechanisms for flagging incorrect outputs, regular audits, and public reporting of accuracy metrics establishing accountability beyond interface design into governance.

#### Pillar Relationships and Implementation Dependencies

While Figure 4 presents the pillars in a circular arrangement emphasizing their interconnectedness during operation, successful implementation requires understanding their dependencies. Pillar 1 (Human-AI Collaboration) serves as the foundational layer with no prerequisites: stakeholders universally prioritized human oversight as nonnegotiable. Pillars 2 (Transparent Logic) and 3 (Contextual Personalization) build upon this foundation and can be developed in parallel, addressing complementary needs: how the AI reasons and how explanations adapt to individual users. Pillar 4 (Cultural-Linguistic Relevance) transforms how existing functionality is expressed within local contexts, requiring the lower pillars to function properly first. Pillar 5 (Trust Calibration and Ethical Safeguards) serves as the governance layer monitoring the complete system, validating that human-AI collaboration follows ethical guidelines, transparent logic reveals true reasoning, personalization respects privacy, and cultural adaptation is authentic. This hierarchical structure informs implementation priorities: establish human oversight first (Pillar 1), build technical and adaptive capabilities (Pillars 2‐3), integrate cultural context (Pillar 4), and implement continuous governance (Pillar 5). We need to provide AI-specific informed consent explaining how algorithms use the GPS, health history, and demographics to generate explanations; data minimization with on-device processing where feasible; user control over personalization settings; and adherence to frameworks, including the European Union’s AI Act’s requirements for high-risk health AI systems [36] and WHO guidance on AI ethics [6]. Continuous monitoring must audit for potential harms, including privacy breaches, discriminatory personalization, and trust miscalibration through regular privacy impact assessments.

#### Framework Synthesis

The framework addresses identified stakeholder misalignments by reconciling users’ need for accessible explanations with clinicians’ demand for evidence-based validation, developers’ feasibility constraints, and experts’ interpretability requirements. These 5 principles form an interconnected system where user experiences refine explanations, clinical validation improves reliability, and contextual adaptation enhances engagement.

## Discussion

### Principal Findings

This study makes 5 principal contributions. First, education level significantly predicted trust in AI explainability (*F*_3, 133_=2.81, *P*=.042), with critical thinking skills mattering more than demographic factors. Second, systematic stakeholder misalignments emerged: users prioritized human validation, developers faced resource constraints limiting XAI implementation, clinicians demanded clinical validation pathways, and experts confirmed technical methods (LIME, SHAP) inadequately serve lay users. Third, all stakeholders universally prioritized human oversight over autonomous AI, validating “social explainability” [8] in health care contexts. Fourth, current technical XAI methods fail to translate into comprehensible explanations for users with varying education levels. Fifth, we developed a five-pillar participatory framework addressing these gaps: (1) human-AI collaboration, (2) transparent logic paths, (3) contextual personalization, (4) cultural-linguistic relevance, and (5) trust calibration with ethical safeguards.

### Interpretation of Statistical Findings

Education level significantly predicted trust in AI explainability (*F*_3, 133_=2.81, *P*=.042, η²=0.060 vs *F*_3, 133_=0.62, *P*=.60), challenging “digital native” assumptions about generational technology adoption differences. The education-trust relationship revealed an unexpected nonlinear pattern. Completed undergraduate students reported lower trust (mean 3.14) than current undergraduates (mean 3.66) and higher secondary students (mean 4.67). This “critical evaluation dip” may reflect heightened critical thinking skills without yet gaining specialized knowledge that contextualizes AI’s appropriate health care role. Postgraduate holders showed higher trust (mean 3.85), possibly reflecting greater AI exposure, and understanding of AI as an assistive rather than a replacement tool. The small effect size (η²=0.060) indicates education accounts for only 6% of variance, suggesting multiple factors influence trust. The absence of age-related differences (*P*=.60) was contrary to expectations. Despite 67.9% of participants being aged 18‐24 years, trust remained consistent across age groups, suggesting explainability requirements are universally important rather than generation-specific. Chi-square analyses reinforced these patterns: education level (*χ*²_6_=17.08, *P*=.009) and XAI familiarity (*χ*²_4_=11.50, *P*=.02) significantly predicted belief in app decisions. Postgraduate holders showed 69% belief rates versus 43% among completed undergraduates. Notably, users “somewhat familiar” with XAI showed the lowest belief (33%), suggesting superficial knowledge increases skepticism without providing sufficient context, indicating explainability education must be comprehensive rather than superficial.

These findings have important design implications. Rather than age-based personalization, mHealth systems should adapt explanations based on educational background and critical thinking orientation. Apps should include educational components explaining what explainability means and why it matters. These quantitative patterns align with qualitative findings, where recent graduates expressed heightened skepticism while postgraduate holders demonstrated more nuanced AI understanding, strengthening confidence in our conclusions.

### Contextual and Relational Explainability

Users appreciated mHealth accessibility but doubted recommendations, citing vague, culturally disconnected outputs. Trust depended on understandable, localized explanations endorsed by professionals, extending prior work [47,51] while highlighting how explainability is reshaped in South Asia, where health literacy and infrastructure vary widely [49,55]. Experts and clinicians suggested that visual explanations and culturally grounded metaphors may be more effective than statistical confidence levels for users with varying literacy levels. These findings parallel Ismail and Kumar’s [56] work with Indian community health workers who require sociocultural context and human mediation, as well as Nigerian/Kenyan digital health, which faces similar infrastructure constraints and language gaps [18,49]. Our framework’s human oversight and cultural adaptation principles appear transferable across South Asian contexts while requiring local implementation adaptation.

### User Focus on Output Explainability Rather Than Technical Implementation

A critical finding emerged from comparing user responses across the 3 apps. Despite Ada using Bayesian reasoning, Symptomate using moderate algorithmic transparency, and WebMD relying on conventional rule-based logic, users showed no significant differences in trust ratings across these apps. This was not a limitation of our study design but rather an intentional research decision that revealed fundamental insights about what users care about in health technology.

We deliberately selected these 3 apps with varying technical architectures to test whether users differentiate based on underlying AI implementation. The results definitely show they do not. Users are not concerned with whether an app uses ML, Bayesian networks, or traditional algorithms. Instead, they evaluate systems based on whether the output is explained clearly and whether it can be validated by trusted human authorities.

This finding directly supports our framework’s design priorities. Users consistently demanded the same things across all 3 apps, regardless of technical implementation: clear explanations of recommendations, understanding of why specific conclusions were reached, and access to doctor validation. Whether the system used sophisticated AI or simple rule-based logic made no difference to users. What mattered was whether they understood the output and could get human confirmation.

This validates our framework’s structure. We positioned Human-AI Collaboration as the foundational first pillar precisely because users prioritize human validation over algorithmic sophistication. The technical architecture is invisible and irrelevant to users. They want to know what the system concluded, why it matters for their health, and whether a doctor agrees. Similarly, our fourth pillar emphasizes Cultural-Linguistic Relevance because users need explanations in familiar terms and local context, not technical details about model architecture.

The lack of differentiation across apps reveals that the AI explainability problem in health care is fundamentally about human communication, not technical transparency. Users do not want to see LIME visualizations or SHAP values. They want plain language explanations and professional endorsement. Our framework addresses this by prioritizing social explainability mechanisms (human validation, clear communication, and cultural adaptation) over purely technical interpretability approaches.

This finding challenges the dominant narrative in XAI research that focuses on making algorithms more transparent. Our results suggest that for health care users, algorithmic transparency is neither necessary nor sufficient for trust. What users need is output transparency combined with human oversight, which is exactly what our framework provides through its hierarchical pillar structure.

### Economic Feasibility of Human-AI Collaboration

Developer stakeholders questioned the financial viability of human-in-the-loop systems, noting that paying clinicians contradicts mHealth’s cost-saving purpose. On the other hand, the users were willing to pay for expert advice. However, multiple business models can be adapted to achieve economic viability.

A tiered service model could offer users choices between paid premium services (immediate doctor consultation) and free government or NGO (nongovernmental organization)-funded validation through layered review (community health workers escalating to physicians) or asynchronous consultation within 24‐48 hours. This freemium approach, where paying users subsidize infrastructure while disadvantaged users access free services, is common in digital health platforms globally and could be adapted for AI-validated mHealth.

Bangladesh Government infrastructure already supports subsidized services. The Ministry of Health received 41,908 crore BDT (US $3.5 billion) in FY2025-26, with 4166 crore BDT (US $350 million) for free services [69]. The Digital Health Strategy operates 96 telemedicine centers [70], and Shasthyo Batayon handled 10 million calls at 3.6 BDT (US $0.0289) per consultation during COVID-19 [71]. WHO, United Nations Children's Fund, United Nations Population Fund [72], World Bank (US $200 million) [73], and NGOs like BRAC [74] provide additional funding streams that could support free validation.

Additional models include subscription-based access, insurance integration, and risk stratification that reserves physician validation for high-risk cases while AI handles low-risk assessments.

### Sociocultural Dimensions and Stakeholder Gaps

Stakeholder expectations diverged: users preferred concise, reassuring messages; clinicians sought traceable logic aligned with guidelines; developers prioritized scalability; and experts stressed ethical clarity. These divergent priorities confirm that explainability must function simultaneously as a cognitive, ethical, and cultural interface.

### Design Trade-Offs and Implications

Developing explainable mHealth AI involves unavoidable trade-offs: simplicity improves comprehension but risks omitting nuance; personalization increases relevance but raises resource demands; transparency can overwhelm low-literacy users; and cultural adaptation strengthens trust but challenges standardization. These tensions necessitate layered, adaptive explanations varying in depth according to user capability and task sensitivity, a design principle embedded in our 5-pillar framework integrating human-AI collaboration, transparent logic, contextual personalization, cultural alignment, and ethical safeguards.

### Practical and Ethical Relevance

The framework’s contribution lies in translating explainability into a responsible design principle. It operationalizes human oversight, transparency, and ethical accountability within mHealth ecosystems where literacy and infrastructure constraints persist. Developers in the study confirmed the feasibility for incremental deployment, starting with foundational safeguards, then progressively adding personalization and cultural adaptation. This approach promotes long-term sustainability while addressing the AI equity gap by enabling inclusive, interpretable, and locally meaningful digital health tools.

### Limitations and Future Directions

#### Sample Representativeness

A critical limitation is the discrepancy between our motivating context (low-resource, underserved populations) and actual participants (predominantly young, highly educated, and English-proficient). This gap exists because evaluated apps (Ada, WebMD, Symptomate) are English-only; Bangla-language mHealth apps with XAI features do not currently exist. This technological constraint necessitated recruiting English-proficient participants representing educated early adopters rather than the general population, rural communities, or low-literacy groups who would benefit most from accessible mHealth solutions. Another limitation concerns sample size imbalance across education categories. The Higher Secondary group contained only 3 participants, while the Undergraduate (running) group had 107 students. Although the ANOVA showed statistical significance (*F*_3, 133_=2.81, *P*=.042), the extremely small Higher Secondary sample means this finding should be considered anecdotal rather than definitive. The more reliable observation is that completed undergraduate students showed lower trust (mean 3.14, SD 1.10) compared with current undergraduates (mean 3.66, SD 0.93), as both groups had adequate sample sizes for comparison. Future research needs larger and more balanced samples across all education levels to confirm whether education truly predicts trust in AI explainability.

#### Selection Bias

Interview and focus group participants (n=20) were recruited through professional networks and snowball sampling, likely selecting technology-interested individuals, and over-representing optimistic perspectives. The “cautiously optimistic” characterization should be interpreted; accordingly, real-world skepticism may be greater than findings indicate. Based on expert and clinician insights, framework applicability to low-literacy populations may require adaptations: visual/audio explanations, oral health traditions, local language dependence, and community health worker intermediaries. Direct validation with low-literacy users is needed. This study was conducted in Bangladesh with educated, urban participants. Findings may not generalize across all South Asian contexts given regional variations in infrastructure, literacy, and cultural practices.

#### Language Barriers

Even among English-proficient participants, English-language interfaces may have acted as trust barriers distinct from AI opacity, reducing cultural resonance and increasing cognitive load. Trust deficits likely reflect both AI design inadequacies and linguistic-cultural barriers. Findings cannot be generalized to illiterate, semiliterate, or non–English-speaking populations whose perspectives remain critically underrepresented.

#### Methodological Limitations

Small subgroup samples (above 40 years [n=4]; higher secondary education [n=3]) limit statistical power. Education’s small effect size (η²=0.060) indicates it accounts for only 6% of trust variance, highlighting the influence of unmeasured factors. The developer sample (n=4) was small; findings represent preliminary insights requiring validation. Qualitative analysis used single-coder coding without independent inter-coder reliability testing, though peer debriefing was used.

Additionally, nearly half of the respondents (48.2%) were first-time users who installed the app immediately before survey completion. Their trust ratings likely reflect initial usability impressions rather than sustained trust developed through repeated use and verification of AI accuracy against actual health outcomes. Future research should examine how trust evolves across multiple usage sessions to distinguish between first-impression reactions and experience-based confidence.

Furthermore, WebMD uses rule-based logic rather than ML, meaning approximately one-third of participants (n≈46) evaluated a traditional algorithmic system rather than AI. Their trust and explainability ratings reflect mHealth interface usability rather than reactions to AI opacity. While we intentionally included WebMD to test whether users differentiate based on technical architecture, this means not all user responses address AI-specific explainability. The lack of differentiation between AI and non-AI systems is theoretically significant, but readers should note that the user sample includes evaluations of both system types.

#### Framework Validation

This framework represents a conceptual model synthesized from stakeholder requirements and existing XAI literature. While grounded in empirical multistakeholder data and theoretically informed by participatory design principles, the framework has not been empirically validated through expert evaluation (eg, cognitive walkthroughs, heuristic evaluation), prototype implementation, or usability testing with end users. Validation through expert review, pilot implementation, and user testing is required before operationalization in real-world mHealth apps to assess whether the framework successfully addresses identified gaps and whether users comprehend and trust explanations designed according to these principles.

#### Future Research Priorities

Urgent needs include (1) developing Bangla-language mHealth apps with culturally adapted XAI for low-literacy populations; (2) participatory design with illiterate, semiliterate, and non–English-speaking users to understand their unique explainability needs; (3) prototype implementation and expert evaluation of the framework; (4) longitudinal research evaluating how culturally tailored XAI influences health outcomes, engagement, and trust over time; (5) cross-cultural validation across diverse Global South as well as South Asian contexts.

#### Comparison With Existing mHealth Apps

To assess current practices, widely used apps like Ada, WebMD, and Symptomate [59] were reviewed. These tools offer basic symptom guidance but lack structured explainability, localized communication, and clinical validation. None incorporates human oversight or clear reasoning paths, often providing opaque or English-only feedback. In contrast, the proposed framework directly addresses these deficiencies through clinician integration, traceable logic, contextual personalization, and ethical transparency. Table 6 summarizes these features.

**Table 6.** Comparison of existing mobile health (mHealth) apps with the proposed 5-pillar framework.

Pillars	Existing apps (Ada, WebMD, Symptomate)	Five-pillar framework
Human-AI^a^ collaboration and clinical validation	No integration with human oversight. All 3 apps are fully automated with no clinician handoff or validation [75-77].	Encourages human-in-the-loop systems, including clinician referrals or expert review during critical decision points.
Transparent logic paths	Opaque decision logic. Ada uses Bayesian networks but does not show reasoning to users; WebMD and Symptomate provide no logic traceability [76-78].	Recommends user-facing, traceable decision paths through visual logic flows, confidence scores, or symptom highlighting.
Contextual personalization	Basic personalization (eg, age, gender). No adaptation to literacy, cultural norms, or prior user behavior [79,80].	Supports layered explanation complexity tailored to digital literacy, history of use, and cultural behaviors.
Cultural and linguistic relevance	Mostly English-based; limited localization. Explanations are generic, lacking cultural alignment [75-77].	Includes native language support, culturally familiar metaphors, and visual storytelling.
Trust calibration and ethical safeguards	Disclaimers are included, but lack detail on model accuracy, limitations, or fairness [81,82].	Emphasizes ethical design with clear disclaimers, data transparency, fairness disclosures, and user control.

a AI: artificial intelligence.

These contrasts underscore the practical value of the framework as a blueprint for improving explainability standards in mHealth. By situating AI explanations within social, cultural, and clinical realities, it offers a model that enhances trust, interpretability, and user safety.

#### Comparison With Existing mHealth Evaluation Frameworks

Table 7 situates our framework within the existing mHealth XAI literature, comparing this study’s approach and contributions to 16 prior works across methodological, theoretical, and practical dimensions.

**Table 7.** Comparison of this study with prior explainable AI (XAI) and mobile health (mHealth) research.

Study	Context and sample	Methods	Key findings	Limitations	How this study advances globally
Kim et al (2024) [83]	South Korea; Clinicians & patients	Scenario-based needs assessment	Clinicians and patients have distinct explanation needs; designed stakeholder-tailored interfaces	Single disease (health management); 2 stakeholder groups only; no developers; controlled scenario	Multistakeholder including developers (who implement XAI); real-world mHealth apps; identifies implementation barriers (resource constraints, lack of tools); findings apply to any 2-sided health platform globally
Health care (MDPI) (2025) [84]	Western context: n=35 (5 experts, 30 users)	Usability evaluation of ADA, Mediktor, WebMD	All apps failed XAI heuristics; no confidence scores or rationales provided; critical transparency gaps	Identifies WHAT gaps exist but not WHY; no developer/clinician input; limited geographic diversity	Explains WHY XAI gaps exist through developer interviews (resource constraints, lack of localized tools); applicable to startups/SMEs^a^ globally
Noor et al (2025) [85]	Global review; 1226 studies (2000‐2023)	Systematic review	Categorized XAI methods (LIME^b^, SHAP^c^, GradCAM); technical taxonomy for health care AI^d^	Technical focus; no mHealth-specific analysis; no user comprehension studies	Validates user comprehension across education levels; demonstrates LIME/SHAP inadequate for lay users universally; education predicts trust (*P*=.042)
MDPI Study (2025) [86]	Global review; recent evidence (2024‐2025)	Narrative review	Shift toward mental health AI; integration challenges identified across contexts	Review only; no actionable framework; no primary data	Provides actionable 5-pillar framework addressing integration challenges; stakeholder misalignments (universal across health care systems)
Doshi-Velez and Kim (2017) [11]	USA; conceptual	Theoretical framework	Defined interpretability evaluation criteria for ML^e^	No empirical validation; assumes universal technical audience	Empirical validation with diverse stakeholders; interpretability needs vary by education (universal), not geography
Rudin (2019) [13]	USA; conceptual	Position paper	Advocacy for inherently interpretable models versus post-hoc explanations	Theoretical; no user studies; model-choice focus	Users across education levels prefer interpretable outputs; validates Rudin’s thesis with empirical data; applicable to high-stakes AI globally
Ribeiro et al (2016) [49] LIME	USA; technical validation	Post-hoc method	LIME algorithm for local feature importance	Developer-centric tool; not designed for end users	Expert/developer consensus: LIME inadequate for lay users universally; technical-lay user gap exists globally
Lundberg and Lee (2017) [50] SHAP	USA; technical validation	Unified interpretation method	SHAP values for game-theoretic feature importance	Technical complexity; assumes mathematical literacy	Developer testimony: “SHAP for developers, not users”; mathematical complexity barrier is universal, not cultural
Liao et al (2020) [53]	USA; 20 practitioners	Qualitative interviews	Design patterns for XAI user experience	Practitioners only; no end users; high-resource assumptions	Extends with end user perspectives (n=137); universal needs (transparency, actionability) transcend geographic boundaries
Larasati et al (2023) [32]	Netherlands; 24 patients	Mixed methods	Trust influenced by explanation meaningfulness	High-resource, Western health care; patients only	Validates trust meaningfulness link across resource contexts; extends to multistakeholder (developers, clinicians, experts)
Visser et al (2023) [12]	Germany; review of 47 studies	Systematic review	Trust depends on explanation quality and user expertise	Geographic bias (45/47 Western); limited demographic analysis	Empirical demographic data; education (not age) predicts trust—likely universal pattern; tests Western assumptions in non-Western context
He et al (2022) [33]	China; user study	User-centered design	User needs vary by medical knowledge level	Single country; limited sample; no framework	Corroborates knowledge-level finding; education-based personalization universal; 5-pillar framework operationalizes insights globally
Rosenbacke et al (2024) [10]	Global review; 22 studies (20 Western)	Systematic review	XAI can increase OR decrease clinician trust depending on design	Severe Western bias; clinicians only; no user perspectives	Non-Western empirical data; validates universal finding: poor XAI decreases trust; clinician-user gap universal across contexts
Shin (2021) [9]	South Korea; 405 users	Quantitative experiment	Explainability enhances trust and perceived control	Not health care-specific; single country	Health care-specific validation; trust-explainability link holds across contexts (universal psychological mechanism)
Okolo et al (2022) [17]	Global South review; 63 papers	Systematic review	Identified severe XAI research gap in Global South	Conceptual; no primary empirical data	Fills identified gap; but findings (education predicts trust, stakeholder misalignments) are universal patterns applicable to underserved populations globally
Sambasivan et al (2021) [48]	India; 67 participants	Ethnographic study	Fairness assumptions from West fail in non-Western contexts	Data work focus; not health care XAI	Extends fairness critique to explainability; Western XAI assumptions (technical transparency sufficient, age predicts trust) fail universally
This Study	Bangladesh; 157 total: Users (n=137), Developers (n=4), Clinicians (n=10), XAI Experts (n=6)	Sequential mixed methods: quantitative surveys, qualitative interviews/focus groups; statistical+ thematic analysis	Five-pillar universal framework: (1) Human-AI collaboration, (2) Transparent logic, (3) Contextual personalization, (4) Cultural-linguistic relevance, (5) Ethical safeguards. Education (not age) predicts trust (*P*=.042). Stakeholder misalignments documented. LIME/SHAP inadequate for lay users.	Single site (but generalizable patterns); English-proficient sample; 4 developers; selection bias	Universal Contributions: (1) Education predicts trust globally, applies to elderly care (Japan/Germany), health literacy programs (worldwide), digital inclusion (everywhere); (2) Stakeholder misalignments exist in ALL health care systems (US/European systems face same gaps); (3) LIME/SHAP inadequacy universal, not context-specific; (4) Framework applicable to ANY context: immigrant populations (US/Europe), Indigenous communities (Australia/Canada), rural populations (all continents), multilingual regions (Southeast Asia, Africa, South America), low-resource settings (rural US, Eastern Europe); (5) Developer constraints (limited resources, lack of tools) affect startups globally; (6) Replicable multistakeholder methodology for any context

a SME: small and medium-sized enterprise.

b LIME: local interpretable model-agnostic explanations.

c SHAP: Shapley additive explanations.

d AI: artificial intelligence.

e ML: machine learning.

### Conclusions

This multistakeholder study identified five principal findings: (1) Education significantly predicts trust in AI explainability (*P*=.042); (2) Systematic misalignments exist between user needs, developer constraints, and clinician requirements; (3) Human oversight is universally prioritized over autonomous AI; (4) Technical XAI methods inadequately serve lay users; (5) A 5-pillar participatory framework addresses these gaps. These findings benefit mHealth developers (implementation guidance for resource-constrained contexts), health care providers (evidence for human-AI collaboration models), policymakers (principles for AI governance and digital literacy programs), XAI researchers (identification of technical-lay user gap), end users (advocacy for understandable health AI), and global health equity (framework preventing Western-centric paradigms and addressing challenges affecting billions worldwide).

Explainability in AI-driven mHealth apps is not merely a question of technical transparency but a social, cultural, and ethical necessity. This study, grounded in the South Asian context, reveals that users, developers, and medical professionals conceptualize explainability in distinct yet interconnected ways: users seek reassurance and clarity, developers emphasize feasibility and performance, and clinicians demand evidence-based accountability. The proposed 5-pillar framework responds to these diverse expectations by integrating human–AI collaboration, transparent logic, contextual personalization, cultural relevance, and ethical safeguards into a unified model for trustworthy mHealth design.

Beyond advancing technical discourse, this work highlights that explainability in health care AI is fundamentally about equity and inclusion. In resource-limited settings, the ability to understand and question AI-generated recommendations determines whether digital tools empower or marginalize users. By situating explainability within local linguistic and cultural realities, this research contributes a context-sensitive foundation for designing AI systems that are not only intelligent but also understandable, credible, and humane.

Ultimately, the study calls for a paradigm shift from viewing explainability as a computational problem to treating it as a participatory design responsibility. Building trustworthy mHealth AI requires more than transparent models; it demands collaboration across disciplines and cultures. The findings and framework presented here aim to inform that transformation, guiding the development of AI systems that strengthen, not replace, the relationship between humans and technology in the pursuit of better health.

## Supplementary material

10.2196/87158 Multimedia Appendix 1 Interview guide for stakeholder interviews with developers, clinicians, and XAI experts.

10.2196/87158 Multimedia Appendix 2 Survey questionnaires administered to end users.

## Abbreviations

AD: application developer
AI: artificial intelligence
KII: Key Informant Interview
LIME: local interpretable model-agnostic explanations
mHealth: mobile health
ML: machine learning
NGO: nongovernmental organization
SHAP: Shapley additive explanations
WHO: World Health Organization
XAI: explainable AI
