# Supplementary material for: A Proposed Participatory Framework for Explainable AI in mHealth: Mixed Methods Study Integrating User and Stakeholder Requirements
Source: J Med Internet Res. 2026 May 4;28:e87158. doi: 10.2196/87158 (PMC13138816; doi:10.2196/87158)
Supplement: Multimedia Appendix 1 [file jmir-v28-e87158-s001.docx]

**Multimedia Appendix 1:**

**Interview Guide for Stakeholder Interviews with Developers, Clinicians, and XAI Experts**

**Introduction**

This semi-structured interview guide was developed to explore stakeholder perspectives on explainable artificial intelligence (XAI) in mobile health (mHealth) applications. Interviews were conducted with three stakeholder groups: mHealth app developers, medical doctors/clinicians, and XAI experts.

The semi-structured format allowed for follow-up questions and exploration of emergent themes while maintaining consistency across interviews. All participants provided informed consent and were assured of confidentiality.

Interview Protocol:

1. Introduction and consent confirmation

2. Background and demographic questions

3. Main interview questions

4. Open-ended closing question for additional insights

5. Thank you and next steps

═══════════════════════════════════════════════════════

SECTION A: INTERVIEW GUIDE FOR AI APP DEVELOPERS

1. Can you describe your role and your familiarity with AI-driven mHealth apps?
2. How do you perceive the role of mHealth apps in improving healthcare services in Bangladesh?
3. What is your thought about AI’s medical capabilities?
4. Do you think mHealth apps provide sufficient explanations for their health recommendations? Why or why not?
5. What aspects of AI explanations do you find most helpful in making informed health decisions (based on user feedback or design perspective)?
6. What factors influence your trust in AI-generated recommendations in health apps (from a development standpoint)?
7. Would a more transparent system (e.g., showing how AI reaches its conclusions) increase user confidence in using these apps? Why or why not?
8. How do you think AI-driven mHealth apps can balance data-driven insights with users’ need for personalized and contextualized explanations?
9. What explainability features would make AI-driven mHealth apps more useful?
10. Are there any privacy concerns regarding AI explaining its decisions, especially in digital healthcare?
11. What type of explanations would you prefer to have for user trust in app suggestions (e.g. visuals, text, graphs, audio, videos)?
12. Are there specific local healthcare challenges (e.g., language barriers, literacy levels, affordability) that AI-driven mHealth apps should address?
13. Do you think AI-driven mHealth apps should include features like live chat with medical professionals to enhance trust and usability?
14. Is there anything else you would like to share about explainable AI in mHealth that we haven't discussed?

═══════════════════════════════════════════════════════SECTION B: INTERVIEW GUIDE FOR MEDICAL DOCTORS/CLINICIANS

1. Can you describe your role and your familiarity with AI-driven mHealth apps?
2. How do you perceive the role of mHealth apps in improving healthcare services in Bangladesh?
3. What is your thought about AI’s medical capabilities?
4. What are the most common health concerns for which you use AI-driven mHealth apps?
5. How familiar are you with AI-generated recommendations in these apps? Do you rely on them for making health-related decisions?
6. Do you think mHealth apps provide sufficient explanations for their health recommendations? Why or why not?
7. Have you ever felt uncertain about an AI-generated health recommendation? Can you describe the situation?
8. What aspects of AI explanations do you find most helpful in making informed health decisions?
9. Have you encountered cases where a lack of trust in AI-driven recommendations influenced your or others' health-related decisions?
10. In your opinion, why might people prefer human healthcare providers over AI-based recommendations?
11. Do you think AI has the medical capability to provide trustworthy recommendations? Why or why not?
12. Do you think human expertise is needed to start medication or treatment? Why and Why not?
13. Have you observed instances where AI-generated recommendations were trusted or dismissed by healthcare professionals or patients? Why?
14. Do cultural beliefs or traditional healthcare practices in Bangladesh influence how people perceive AI-driven health recommendations?
15. Are there specific local healthcare challenges (e.g., language barriers, literacy levels, affordability) that AI-driven mHealth apps should address?
16. Would you be more likely to trust an AI system if it was endorsed by Bangladeshi medical institutions or healthcare professionals?
17. Do you think AI-driven mHealth apps should include features like live chat with medical professionals to enhance trust and usability?
18. Is there anything else about AI in healthcare or mHealth apps that you think is important for us to understand from a clinical perspective?

═══════════════════════════════════════════════════════SECTION C: INTERVIEW GUIDE FOR XAI EXPERTS

1. Can you describe your role and your familiarity with AI-driven mHealth apps?
2. What is your thought about AI’s medical capabilities?
3. Do you think mHealth apps provide sufficient explanations for their health recommendations? Why or why not?
4. What aspects of AI explanations do you find most helpful in making informed health decisions (from a user or design point of view)?
5. What factors influence user trust in AI-generated recommendations in health apps?
6. Would a more transparent system (e.g., showing how AI reaches its conclusions) increase user confidence in using these apps? Why or why not?
7. How do you think AI-driven mHealth apps can balance data-driven insights with users’ need for personalized and contextualized explanations?
8. What explainability features would make AI-driven mHealth apps more useful?
9. Are there any privacy concerns regarding AI explaining its decisions, especially in digital healthcare?
10. What type of explanations would be most effective in building trust (e.g. visuals, text, graphs, audio, videos)?
11. Have you observed instances where AI-generated recommendations were trusted or dismissed by healthcare professionals or patients? Why?
12. Do you think AI-driven mHealth apps should include features like live chat with medical professionals to enhance trust and usability?
13. From an XAI perspective, what do you think are the most critical considerations for implementing explainable AI in mHealth apps in resource-constrained settings like Bangladesh?

═══════════════════════════════════════════════════════END OF INTERVIEW GUIDE

Note: This interview guide was used flexibly to allow for natural conversation flow and exploration of emergent themes. Interviewers used follow-up probes such as "Can you tell me more about that?", "Can you give an example?", and "Why do you think that is?" to elicit detailed responses.
