# Supplementary material for: A Proposed Participatory Framework for Explainable AI in mHealth: Mixed Methods Study Integrating User and Stakeholder Requirements
Source: J Med Internet Res. 2026 May 4;28:e87158. doi: 10.2196/87158 (PMC13138816; doi:10.2196/87158)

Multimedia Appendix 2:

Survey Questionnaires Administered to End-users

This questionnaire was administered using Google Forms. Below is a transcription of the questions included in the survey.

1. Please specify your gender.

*(Multiple choice question)*

- - Male
  - Female
  - Other

1. What is your age group?

*(Multiple choice question)*

- 18–24

- 25–30

- 31–40

- Above 40

1. Please mention your hometown.

*(Dropdown)*

- Bagerhat
- Bandarban
- Barguna
- Barishal
- Bhola
- Bogura
- Brahmanbaria
- Chandpur
- Chattogram
- Chuadanga
- Chapai Nawabganj
- Cox’s Bazar
- Cumilla
- Dhaka
- Dinajpur
- Faridpur
- Feni
- Gaibandha
- Gazipur
- Gopalganj
- Habiganj
- Jamalpur
- Jashore
- Jhalokathi
- Jhenaidah
- Joypurhat
- Khagrachari
- Khulna
- Kishoreganj
- Kurigram
- Kushtia
- Lakshmipur
- Lalmonirhat
- Madaripur
- Magura
- Manikganj
- Meherpur
- Moulvibazar
- Munshiganj
- Mymensingh
- Naogaon
- Narail
- Narayanganj
- Narsingdi
- Natore
- Netrokona
- Nilphamari
- Noakhali
- Pabna
- Panchagarh
- Patuakhali
- Pirojpur
- Rajbari
- Rajshahi
- Rangamati
- Rangpur
- Satkhira
- Shariatpur
- Sherpur
- Sirajganj
- Sunamganj
- Sylhet
- Tangail
- Thakurgaon

1. What is your current/highest education level?

*(Multiple choice)*

- - Primary (1–5)
  - Secondary (6–10)
  - Higher Secondary (11–12)
  - Undergraduate (completed)
  - Undergraduate (running)
  - Postgraduate (Master’s/Doctoral)
  - No formal education

1. Do you use a smartphone?

*(Yes/No question)*

- - Yes
  - No

1. Do you use internet on your smartphone?

*(Yes/No question)*

- - Yes
  - No

1. Do you have any idea about artificial intelligence (AI)?

*(Multiple choice)*

- - Yes
  - No
  - Somewhat familiar

1. Do you use any health-related AI apps on your phone?

*(Multiple choice)*

- - Yes
  - No
  - Maybe / Not sure

1. At the beginning of this survey, we mentioned specific health apps (Ada, Sympto- mate, WebMD). Which app did you actually use? *(Multiple choice)*
   - Symptomate
   - Ada
   - WebMD
2. Upload a screenshot from the app showing your symptom check results.

*(Upload button)*

1. How frequently do you use that app?

*(Multiple choice)*

- - Regularly
  - Sometimes
  - Very rarely

1. Are you familiar with the term explainable AI (known as XAI)?

*(Multiple choice)*

- - Yes
  - No
  - Somewhat familiar

1. What is explainable AI (XAI)?

Before moving forward, we’d like to explain what an explainable AI is: It helps users understand why an AI made a specific decision to trust the process. When a health app gives you advice, Explainable AI means:

- - The app tells you why it gave that advice
  - You can understand how it reached that conclusion
  - You know which symptoms led to the recommendation

Example: Instead of just saying “You might have flu,” an explainable app would say: “You might have flu because you reported high fever (101°F), body aches, and fatigue; these are common flu symptoms.”

1. The app I am using is explainable enough to trust the process. How do you rate this statement?

*(5-point Likert scale)*

- - Strongly Disagree (1)
  - Disagree (2)
  - Neutral (3)
  - Agree (4)
  - Strongly Agree (5)

1. Do you believe the decision/prediction/recommendation made by this app?

*(Multiple choice + short answer)*

- - Yes — Why:
  - No — Why:
  - Not Sure

1. Will you trust this app’s result over your physician/doctor?

*(Multiple choice + short answer)*

- - Yes — Why:
  - No — Why:
  - Not Sure

1. Will you start taking medication/guidance as per this app’s suggestion without further consultation with doctors?

*(Multiple choice + short answer)*

- - Yes — Why:
  - No — Why:
  - Not Sure

1. Any suggestions about this app which can make the app’s AI-based decision more reliable/trustworthy/explainable/reasonable?

*(Open-ended)*


Supplement: Multimedia Appendix 2 [file jmir-v28-e87158-s002.docx]
